# Anhedonia influences threat avoidance and relief: A conceptual replication

**DOI:** 10.1016/j.xjmad.2024.100050

**Published:** 2024-01-17

**Authors:** Lu Leng, Tom Beckers, Bram Vervliet

**Affiliations:** a Center for the Psychology of Learning and Experimental Psychopathology, KU Leuven, Belgium; b Laboratory of Biological Psychology, KU Leuven, Belgium; c Leuven Brain Institute, KU Leuven, Belgium

**Keywords:** Threat avoidance, Relief, Anhedonia

## Abstract

Active threat avoidance is an adaptive coping strategy but can evolve into maladaptive behavior patterns when it is disproportionate to an actual threat. While excessive and persistent avoidance, as often seen in anxiety-related disorders, have been investigated extensively, it is presently unclear under what circumstances insufficient avoidance might occur in the presence of a genuine threat. We hypothesized that anhedonia, the reduced ability to experience pleasure, might undermine the relief experience after successful threat avoidance and thus reduce future active avoidance responses. Using an established avoidance learning paradigm, we examined the relationship between anhedonia, relief, and active avoidance responses. Forty participants learned that two threat cues signaled electrical stimulation and they could click a button during cue presentations to prevent electrical stimulation from occurring. While clicking the button worked for one threat cue, it did not work for the other one. After several repetitions, button effectiveness was reversed. Another safety cue that never signaled electrical stimulation was presented intermixed with the two threat cues. Every time there was an omission of electrical stimulation, self-reported relief was measured. We found that participants who scored higher on anhedonia experienced weaker relief during all outcome omissions. Behaviorally, at the early stage of each phase, participants who scored higher on anhedonia executed fewer avoidance actions, specifically for the threat cue that signaled avoidable electrical stimulation. Relief induced by threat omission is a pleasant experience, which trait anhedonia seems to impair. This attenuation of relief might reduce the reinforcement of future adaptive avoidance behaviors.

## 1 Introduction

Active threat avoidance helps to shield us from harm, which makes it an adaptive coping behavior in the face of impending threats [1]. However, this adaptive coping strategy can evolve into a maladaptive behavior pattern when out of proportion with the actual degree of threat. On the one hand, excessive and persistent avoidance behaviors, as often seen in anxiety-related disorders, interfere with important daily-life activities and block opportunities to update threat beliefs when the threat is no longer present [2]. On the other hand, the opposite end of the avoidance continuum may also be regarded as maladaptive [3] - insufficient active avoidance implies needless and uncontrolled threat confrontations, which could be associated with a feeling of learned helplessness [4]. While excessive and persistent avoidance has received a lot of research attention (for reviews, see [1], [5], [6], [7], it remains largely unclear under what circumstances insufficient avoidance could arise in the presence of a real threat.

In the laboratory, active threat avoidance can be investigated through active avoidance learning tasks, in which one stimulus (conditional stimulus, CS+; e.g., a neutral picture) reliably signals a threat (unconditional stimulus, US; e.g., electrical stimulation) and a predetermined response during the CS+ presentation (active avoidance; e.g., button pressing) prevents that US from occurring. These tasks have been employed to study avoidance profiles of individuals with clinical anxiety, as excessive active avoidance is recognized as a primary symptom of anxiety-related disorders [8]. In fact, individuals with subclinical anxiety [9], as well as individuals with anxiety-related disorders [10] have been shown to exhibit excessive and persistent avoidance responses. Studies in this field have greatly advanced our knowledge of potential pathways (i.e., increased threat appraisal, enhanced threat avoidance tendencies) from adaptive to excessive active avoidance [6], [11] and reducing excessive active avoidance is a crucial element of the treatment of the choice for anxiety-related disorders [12], [13]. In contrast, the other extreme of avoidance – whether and why people would avoid insufficiently, has hardly been investigated.

Leng and colleagues [14] provided the first empirical evidence of insufficient active threat avoidance in people with anhedonia – a loss of interest or pleasure in previously rewarding activities. In an online experiment in a community sample (*N* = 200), participants first learned that two threat cues signaled the presentation of aversive pictures. In the next phase, they could press a button when they saw these threat cues to prevent the aversive pictures from appearing on the screen. While the button pressing worked for one threat cue (CS+ signaling avoidable US, CS+av), it did not work for the other one (CS+ signaling unavoidable US, CS+unav). In the final phase, the button pressing became effective in preventing the US for the previous CS+unav and ineffective for the previous CS+av (instrumental reversal learning). As expected, participants with higher levels of anhedonia experienced attenuated relief at successful omissions of aversive pictures, and they also executed fewer responses to prevent aversive pictures from occurring [14].

According to the avoidance-relief theory [15], omissions of aversive events following active avoidance are thought to elicit a positive feeling of relief, which serves to reinforce the foregoing active avoidance response. In support of this theory, it has been shown that experiencing threat-omission-induced relief shares important characteristics with receiving rewards [16]. Since anhedonia is known to be associated with impairments in reward responding [17], it is not surprising that the pleasant feeling of relief induced by threat omission is attenuated by anhedonia. As a result, the reinforcement by relief of active avoidance responses is attenuated, resulting in weaker active avoidance learning. In line with these arguments, it has been suggested that anhedonia may indeed blunt hedonic responses to the absence of threats [18], which rely on the same mesolimbic circuit that is involved in reward processing [19], [20], [21], [22].

As a transdiagnostic feature to many disorders [23], [24], [25], anhedonia is an important construct to understand as it largely affects the Positive Valence System [26], [27] proposed by Research Domain Criteria [28] – “*primarily responsible for responses to positive motivational situations or contexts, such as reward seeking, consummatory behavior, and reward/habit learning*”. What’s worse, anhedonia obstructs the treatment process in various ways when people who suffer from it do seek for help [29]. As expected, anhedonia is receiving mounting interest in preclinical and translational research as well as in clinical treatments [18], [30] and has been considered a treatment target for various disorders - e.g., anxiety [31], depression [29], and bipolar disorders [32]. Interestingly, recent transdiagnostic treatments specifically targeting anhedonia improved not only the positive outcomes, but also the negative outcomes [33], [34], [35]. These observations raise puzzles and indicate that positive and negative systems might interact in a more complex manner. For instance, increasing positive affect has been suggested to be a viable strategy to enhance learning of fear extinction [36]. In spite of the fact that anhedonia is well-recognized for degrading reward processing, little is known about how anhedonia may impact the reward processing in the threat contexts (i.e., threat-omission-induced relief).

The current study attempted to conceptually replicate the effect of anhedonia on active avoidance learning observed in the previous study [14] while trying to overcome several limitations of that investigation. First, US aversiveness might have been less strong than intended. The aversive pictures used as USs, which were the same across participants, might have been perceived very differently by different individuals, resulting in variable learning rates and relief pleasantness at US omissions. In fact, conducting the experiment online, there was no guarantee that participants would be consistently exposed to these USs as intended rather than looking away at times during US presentations. Here, we individualized the US intensity to make sure the USs used in the task were similarly aversive across participants. Also, USs were unavoidable except through the designated experimental avoidance action. Second, relief was measured exclusively by self-report. It has been shown that threat-omission-induced relief is an emotional state that can be captured via physiological responses as well [37], [38]. In the current study, we therefore added a physiological read-out of relief to supplement verbal relief-pleasantness ratings. Finally, the Temporal Experience of Pleasure Scale used to measure anhedonia may not have been ideal, as some of its items have been suggested to be too vague or culture-specific [39]. We therefore adopted a different anhedonia scale in the present study.

To sum up, the current study employed the same basic paradigm as used in the previous study [14] but with a more potent threat (i.e., electrical stimulation) and additionally included psychophysiological measures to gain a fuller understanding of the relief experienced during active avoidance learning. Also, a different scale was used to measure anhedonia. Consistent with the previous findings, we expected higher levels of anhedonia to be associated with lower relief pleasantness at threat omissions and with reduced active threat avoidance. We further expected higher levels of anhedonia to be associated with reduced psychophysiological response at threat omissions. We followed the original experimental design where an additional reversal learning phase was included to increase uncertainty during the task and maximize our chances of detecting anhedonia-related effects. Consistent with the original study, we expected similar anhedonia-related effects before and after the contingency reversal.

## 2 Materials and methods

### 2.1 Participants

Forty-one participants were recruited and asked to give informed consent before the experiment. Participants received 8 euros or course credit for their participation. Data from one participant were lost due to equipment malfunction. All the analyses were performed on the pre-registered sample of 40 participants (7 males; *M*_age_ = 20.80, *SD*_age_ = 3.28, range = 17–29 years) who were all right-handed. The study protocol was approved by the Social and Societal Ethics Committee of KU Leuven. The pre-registered design, hypotheses, and analysis plan are available at https://osf.io/r2kz7.

### 2.2 Stimuli and design

#### 2.2.1 Stimuli

The conditional stimuli (CSs) were pictures of an office room with a desktop lamp that could light up in the color red, blue, or yellow (adapted from [40], presented against a black background at the middle of the computer screen. The unconditional stimulus (US) was a mild 2-ms electrical stimulus delivered to the top of the wrist of the left hand via two stainless steel electrodes (8-mm diameter; 30-mm inter-electrode distance; filled with K-Y Jelly) from a stimulating bar electrode (Digitimer, Hertfordshire, UK). The stimulation was generated by a Digitimer DS7A stimulator (Digitimer, Hertfordshire, UK). The intensity (milliampere, mA) of the stimulation used in the task was self-selected by the participant via a gradual work-up procedure. Specifically, the participant was instructed that the intensity would start from a very low level (2 mA) and increase in small steps. Participants were asked to rate the intensity on a scale of 0 to 10 where 0 represents “*I feel nothing*” and 10 represents “*This is the maximum pain that I can tolerate*”. Participants were instructed to choose an intensity that is “*highly uncomfortable but not painful*” to them.

All trials started with the presentation of the office room where a lamp lighted up in one of the three colors for 6 s. During the avoidance learning phase, a red button (avoidance cue) was presented at the upper left corner of the CS picture 1 s after the lamp color onset for 2 s. After the avoidance cue offset, the office room with the lighted lamp continued to be presented for another 6 s. The US was delivered at CS offset if the trial was supposed to be paired with a US. Trials were separated by random 12–18 s inter-trial intervals.

#### 2.2.2 Skin conductance measurement and pre-processing

The skin conductance signal was recorded on a BIOPAC MP160 Data Acquisition System using the EDA100D module (BIOPAC, USA). Data was continuously recorded into BIOPAC AcqKnowledge software at 2000 Hz for offline analysis. No online hardware or software filtering was applied. The skin conductance signal was recorded using two disposable and pre-gelled 8-mm Ag/AgCl snap electrodes (Biopac Systems, Goleta, California) with an inter-electrode distance of approximately 10 mm, which were attached on the palm of the left hand.

Raw skin conductance data were pre-processed and analyzed using the Ledalab software package (Ledalab, V3.4.9; http://www.ledalab.de) implemented in Matlab (R2019b, MathWorks Inc., Natick, MA, USA). Artifacts (rapid-transient peaks or drops caused by loose electrodes, movement of the participants, etc.) in the raw data were visually screened and corrected within Ledalab. After that, the data were low-pass filtered at 5 Hz (Butterworth, first-order), downsampled to 100 Hz in Ledalab, and then split into different segments for different experiment phases. Each segment was analyzed separately.

Data analysis was performed using Continuous Decomposition Analysis (CDA) in Ledalab with two optimization runs to improve the fit and reduce the error of the model [41]. CDA applies a deconvolution approach to separate phasic activity from the underlying tonic component. Event-related skin conductance responses (SCRs) based on the event markers were calculated as the time integral of the deconvoluted phasic activity (Integrated SCR, ISCR) within 1 to 4 s after the event (μS*s) with a default minimal amplitude threshold of 0.01 μS. Specifically, the time window was defined as 1–4 s following the CS onset (during the fear acquisition phase, see Experimental procedure below) or following the avoidance cue offset (during the avoidance learning phase) for the CS-anticipatory SCRs and 1–4 s following the US omission/stimulation for the omission SCRs. The SCRs were first square-root transformed to reduce the skewness of the distribution [42] and then range-corrected by Z transformation within individuals to account for interindividual variability in responsiveness [43].

As preregistered, two non-responders (not showing valid SCRs to over three-quarters of the US during the fear acquisition phase) and ten non-learners of SCR (see exclusion criteria below) were identified and their data were excluded for the SCR-related analyses, resulting in a smaller sample size (*N* = 28) for SCR-related analyses. Notably, similar results (see Supplementary Material) were obtained when including these non-learners for hypothesis testing.

#### 2.2.3 Outcome measures

##### 2.2.3.1 Avoidance response

Avoidance response was coded as 1 whenever there was a successful button press during a given trial, otherwise this was coded as 0. Any click outside of the red button or outside of the avoidance response window was treated as unsuccessful avoidance responses, therefore was coded as 0.

##### 2.2.3.2 US expectancy

During each CS presentation, participants rated their expectancy of a US (“*To what extent do you expect an electrical stimulus now?*”). using a scale presented below the CS from 0 (“*certainly no electric stimulus*”) to 10 (“*certainly an electric stimulus*”).

##### 2.2.3.3 Relief pleasantness

Using a computerized Visual Analogue Scale (VAS, 0–100), participants rated the relief pleasantness at the end of trials where a US was omitted (“*How pleasant was the relief that you just felt?*) from ”*neutral*” to “*very pleasant*”.

##### 2.2.3.4 US unpleasantness

Participants rated the US unpleasantness via VAS (0−100) at the end of trials where a US was delivered (“*How unpleasant was the stimulation that you just felt?*”) from “*neutral*” to “*very unpleasant*”.

##### 2.2.3.5 CS valence and arousal

The valence (“*How pleasant do you find this figure?*”) and arousal (“*How stimulating do you find this figure?*”) of each lighted lamp picture were measured via VAS (0−100) from either “*very unpleasant*” for valence or “*passive/calm*” for arousal to either “*very pleasant*” for valence or “*active/exciting*” for arousal during the task.

##### 2.2.3.6 Post-experimental questions

At the end of the experiment, participants reported on VAS (0−100) their (1) general avoidance intention, (2) the general pleasantness of US-omission-induced relief, and (3) the general unpleasantness of the USs. Specifically, we asked: (1) “*During the task, how much did you want to avoid the electrical stimulation?*” Responses were registered from “*not at all*” to “*very much*”; (2) “*If you successfully avoided an electrical stimulation, how pleasant did you feel about the relief?*” Responses were registered from “*neutral*” to “*very pleasant*”; (3) “*How unpleasant was the electrical stimulation for you now?*” Responses were registered from “*neutral*” to “*very unpleasant*”.

#### 2.2.4 Questionnaires

##### 2.2.4.1 Anhedonia

The Snaith Hamilton Pleasure Scale (SHAPS) is a self-report questionnaire used to assess hedonic tone or its absence, anhedonia [44]. The 14-item instrument uses a four-point Likert scale ranging from 1 (“*totally agree*”) to 4 (“*disagree completely*”), with higher total scores indicating severer anhedonic symptoms. The Dutch version of the SHAPS was used in the current study [45].

##### 2.2.4.2 Depression symptoms

The Quick Inventory of Depressive Symptomatology-Self Report (QIDS-SR16) is a self-report measure of depressive symptoms [46]. It has 16 items with higher scores indicating greater severity of depressive symptoms. The official Dutch translation of the Quick Inventory of Depressive Symptomatology self-report was used (translation details are provided on the website: www.ids-qids.org).

Given that anxiety-related traits have been found to correlate with the active avoidance learning process, we also measured trait anxiety, intolerance of uncertainty, and distress tolerance in the current study to control for their effects. The State and Trait Anxiety Inventory - Trait Version (**STAI-T**, 20 items; [47]) was used to assess trait anxiety. The Dutch version by van der Ploeg (1982) was used. The Intolerance of Uncertainty Scale (**IUS**, 27 items; [48])was used to measure how individuals may react to uncertainty. The Dutch version [49] was used. The Distress Tolerance Scale (**DTS**, 15 items; [50]) was used to measure the perceived ability to tolerate emotional distress. Since there is no authorized and validated Dutch translation available, an ad hoc translation was used. The internal consistency for all the used questionnaires is reported in Table 1.

**Table 1:** Means, standard deviations, ranges, and correlations between measures.

				Correlations						
	*α*	*M* (*SD*)	Range	1	2	3	4	5	6	7
1. SHAPS	0.81	21.05 (4.76)	14-32	—						
2. QIDS	0.74	6.35 (3.93)	0-16	0.26		—				
3. STAI-T	0.92	40.08 (11.59)	25-68	0.26	0.84***	—				
4. IUS	0.89	61.58 (14.15)	36-90	0.24	0.57***	0.64***	—			
5. DTS	0.89	3.83 (0.61)	2.47-4.67	-0.11	-0.62***	-0.74***	-0.67***	—		
6. AI	—	83.96 (14.89)	55.37-100	-0.16	-0.08	-0.02	-0.04	-0.01	—	
7. GR	—	73.18 (21.60)	26.85-99.81	-0.45	-0.22	-0.17	-0.20	0.09	0.68***	—
8. GUS	—	73.33 (19.92)	31.48-99.44	-0.33	-0.23	-0.13	-0.07	0.02	0.48	0.60**

Note. SHAPS = The Snaith Hamilton Pleasure Scale; QIDS = Quick Inventory of Depressive Symptoms; STAI-T = The State and Trait Anxiety Inventory - Trait Version; IUS = Intolerance of Uncertainty Scale; DTS = Distress Tolerance Scale; AI = avoidance intention; GR = general relief pleasantness; GUS = general US unpleasantness. *α* = Cronbach's alpha indicating the internal consistency. Multiple corrections were performed using Bonferroni correction. *** *p* < .001.

### 2.3 Experimental procedure

After giving their informed consent and being screened for the exclusion criteria, participants filled in a few demographic questions (gender, age, education level, and mental health status) and completed the questionnaires. Then, the electrodes for skin conductance recordings and electric stimulation were attached.

After the shock calibration procedure, the computer task began, which consisted of three phases: a fear acquisition phase, an active avoidance learning phase, and a reversed active avoidance learning phase. Before starting, the office room with a different color lamp was presented for the valence and arousal ratings. Then, the fear acquisition phase started, which consisted of 4 CS+1, 4 CS+2, and 8 CS- trials. The three colors were randomly selected to be CS+1, CS+2, and CS-. While presentations of CS+1 and CS+2 were always paired with the US, CS- presentation was never accompanied by the US. The fear acquisition phase was divided into two blocks that were counterbalanced among participants (first block: 4 CS+1, 4 CS-; second block: 4 CS+2, 4 CS-). The first and the last trial in each block was always a CS+ trial to facilitate fear acquisition [51]. The rest of the trials were pseudo-randomized within blocks so that there were no more than two same trials presented in a row. Participants were told that ‘*In this task, you will first see a picture of one office room, in which the lamp on the desk lights up in different colors for a few seconds. Following the lighted lamp picture, you may or may not feel an electrical stimulation. If you feel an electrical stimulation, try to find out if there was a pattern*.’ After the fear acquisition phase, each color lamp was presented again for the valence and arousal ratings.

Afterward, the active avoidance learning phase started, where participants were told that ‘*In the following phase, you will perform a similar task again. But now a red button will appear for 2 s after the lamp lights up. During the presentation of this red button, you can click on the red button using the left button of the mouse. Clicking the button may or may not prevent the electrical stimulation afterward, and this may be different for each lamp color*’. In reality, both CS+1 and CS+2 were followed by the US if there was no avoidance response (button clicking). Clicking the button only prevented the US for CS+1 (CS+ signaling avoidable US, CS+av), not CS+2 (CS+ signaling unavoidable US, CS+unav). Clicking anywhere other than the red button had no impact. Without any indication, the reversed active avoidance learning phase then started, where the contingency of avoidance response-US omission was reversed. Clicking the button now canceled the US following CS+2 (now CS+av) but not CS+1 (now CS+unav). During both avoidance learning phases, the CS- was never followed by the US regardless of the avoidance response. Both avoidance learning phases consisted of 8 blocks with one CS+av, one CS+unav, and one CS- in each block, within which the order was randomized.

**Fig. 1:**
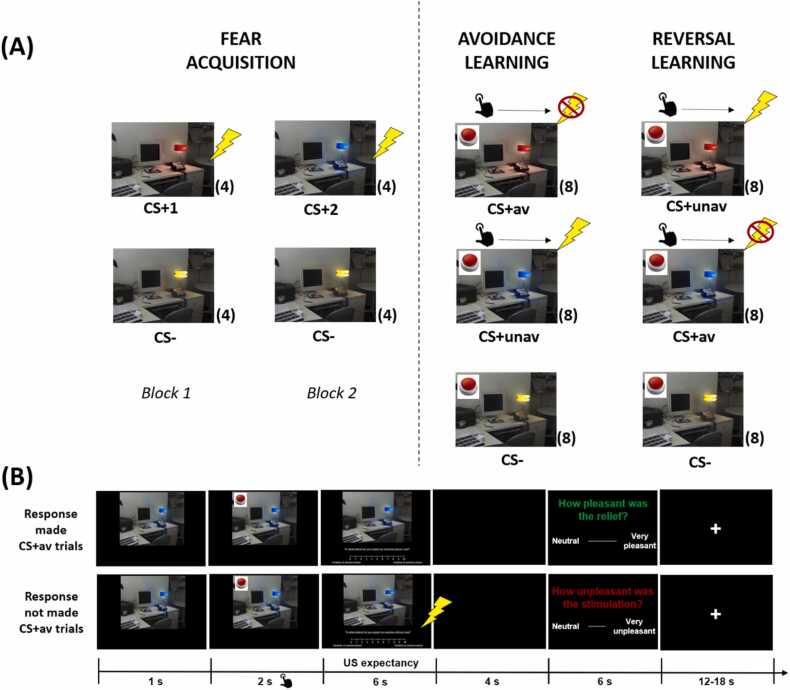
**Experimental design.** (A) The task consisted of three phases, a fear acquisition phase, an avoidance learning phase, and a reversal learning phase. Two color lamps were paired with electrical stimuli during the fear acquisition phase, one of which became the CS+ signaling avoidable US (CS+av) while another became the CS+ signaling unavoidable US (CS+unav) during the following avoidance learning phase. During the reversal learning phase, the previous CS+av signaled unavoidable US (now CS+unav) while the previous CS+unav signaled avoidable US (now CS+av). Clicking on the avoidance cue could cancel the US paired with the CS+av but not the CS+unav. Clicking on the avoidance cue was unnecessary for the CS- since it was never paired with a US during any phases. (B) Overview of the timeline of a CS+av trial with a successful avoidance and a CS+av trial with no avoidance. All trials started with the CS onset and the red button appeared 1 s after that for 2 s. The US-expectancy scale was presented at the time of the red button offset for 6 s. Then, the screen became blank for 4 s if it was a CS+av trial with successful avoidance or a CS- trial, and a scale for the relief pleasantness was presented afterwards for 6 s. An electrical stimulus was delivered if it was a CS+av trial without successful avoidance or a CS+unav trial and a scale for the US unpleasantness was presented afterwards for 6 s. CS+av = the CS+ signaling avoidable US; CS+unav = the CS+ signaling unavoidable US.

Throughout the experiment, the US-expectancy rating scale was presented at the bottom of the screen on every trial for 6 s, starting from the moment of CS onset (during the fear acquisition phase) or the moment of avoidance cue offset (during the two avoidance learning phases). During this 6-s period, participants were asked to indicate their US expectancy. The US was delivered at CS offset when applicable (CS+unav trials or CS+av trials without avoidance response); if so, 4 s later, the rating scale for US unpleasantness was presented for 6 s. Otherwise, the screen stayed blank for 4 s and the rating scale for relief pleasantness was presented on the screen afterward for 6 s. At the end of the task, the post-experimental questions were asked. The experimental design and two example trial flows during the avoidance learning phases can be found in Fig. 1.

## 3 Data processing and analyses

Raw data was first preprocessed and further analyzed in R using RStudio [52]. The original anonymized data and R scripts used in the current study are available at https://osf.io/t78dy/. Main analyses for hypothesis testing were only conducted on data from participants with successful fear acquisition (for US-expectancy-related analyses: either higher US-expectancy ratings on average towards both the CS+1 and the CS+2 compared to the CS- across each block or higher US-expectancy ratings towards the last CS+1 and the last CS+2 compared to the last CS- in each block; for SCR-related analyses: either higher SCR on average towards both the CS+1 and the CS+2 compared to the CS- in each block or higher SCR towards the last CS+1 and the last CS+2 compared to the last CS- in each block).

Linear mixed-effects models (LMM) were used for the hypothesis testing regarding relief experiences and Generalized linear mixed-effects models (GLMM) were used for the hypothesis testing regarding avoidance behaviors (binary responses). All categorical predictors (e.g., Phase, CS) were coded using sum to zero contrasts while continuous predictors (e.g., Trial) were grand-mean centered to make interpretations easier and to reduce multicollinearity when an interaction term was included [53]. A random intercept over all participants was included to account for the repeated-measures nature of the data. Random slopes were included if they improved model fit. The model with the best goodness-of-fit was selected based on the likelihood-ratio test. The detailed model fitting procedure, which followed the previous study [14] and deviated slightly from the procedure as preregistered, is reported in the Supplementary Material. For main hypothesis testing, the main effects of other covariates of no interest (age, gender, US unpleasantness, avoidance intention, STAI-T, IUS, DTS, QIDS) were added into the final model one at a time to test their effects and to check the robustness of our findings. These results can be found in the Supplementary Material.

### 3.1 Fear acquisition

To evaluate whether participants successfully acquired conditioned fear, the US-expectancy ratings and anticipatory SCRs for the 8 CS- trials were averaged per two trials to yield 4 CS- data points, to be compared to the 4 data points for the CS+1 and the CS+2. Then, two sets of LMM models were built with US-expectancy ratings and anticipatory SCRs as outcome variables, respectively. In each set of models, CS (CS+1, CS+2, CS-), Trial, and their interaction were added as fixed-effect factors. Finally, the best model was used to assess fear learning across CS types over trials. Additionally, CS-valence and CS-arousal ratings measured before and after the fear acquisition phase were analyzed using 3 (CS: CS+1, CS+2, CS-) * 2 (Time: before, after) Repeated Measure Analysis of Variance (RM-ANOVA). Greenhouse-Geisser corrections were applied where Mauchly’s test of sphericity was not met. Bonferroni correction was applied to all post-hoc tests to protect against inflated type I errors. Effect sizes are reported as partial eta-squared and the alpha level was set to *α* = 0.05.

### 3.2 Avoidance learning

The avoidance learning process was first examined with the main effects of Phase (Avoidance learning/Reversal learning), CS (CS+av, CS+unav, CS-), and Trial on each trial-wise outcome (avoidance response, US expectancy, anticipatory SCR, relief pleasantness, omission SCR). Since US was always delivered on a CS+unav trial, there was neither relief pleasantness nor omission SCR registered for this trial type. As a result, the factor CS had two levels with these variables being the outcome. We additionally included whether the participant pressed the red button during each trial (variable Avoided with value 1 for a correct avoidance response, i.e., Response made, and value 0 for no or incorrect avoidance response, i.e., Response not made) as a predictor for US expectancy and anticipatory SCR.

### 3.3 Main hypothesis testing

To test the first hypothesis (*higher levels of anhedonia are associated with less relief*), SHAPS was added in the two avoidance learning models with relief pleasantness and omission SCR as the outcome variables, respectively. To test the second hypothesis (*higher levels of anhedonia are associated with less active avoidance*), SHAPS was added in the avoidance learning model with avoidance responses as the outcome variable.

## 4 Results

### 4.1 Self-reported measures and correlations

The internal consistency, mean, standard deviation, and range of the questionnaire scores and the post-experimental ratings (Avoidance Intention, General Relief, General US unpleasantness) are summarized in Table 1. Spearman correlation was used since most of the variables failed to conform to a normal distribution.

### 4.2 Fear acquisition and avoidance learning effects

**Fig. 2:**
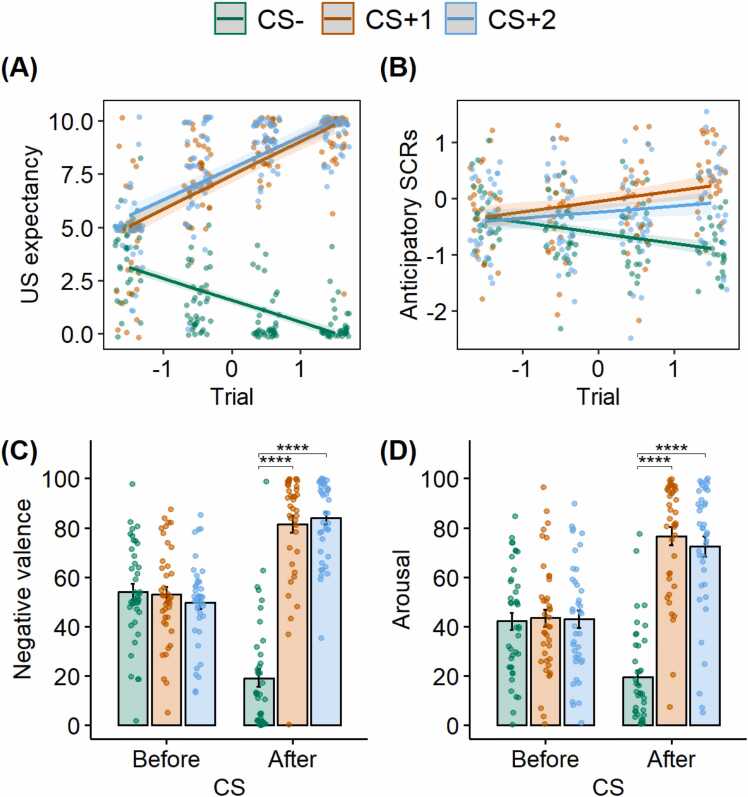
**Successful fear acquisition indicated by different measures.** (A) US-expectancy ratings ranged from 0 to 10, where 0 represented “*certainly no electric stimulus*” and 10 represented “*certainly an electric stimulus*”; (B) Anticipatory SCR, which was square-root transformed and standardized; (C) Negative valence ratings ranged from 0 to 100, where 0 represented “*very pleasant*” and 100 represented “*very unpleasant*”; (D) Arousal ratings ranged from 0 to 100, where 0 represented “*passive/calm*” and 100 represented “*active/stimulating*”. CS+1 = the CS+ in the first block; CS+2 = the CS+ in the second block. Dots represent individual participant data. Error bars represent standard error of the mean. **** *p* < .0001.

More detailed results for this section can be found in the Supplementary Material. To summarize, during the fear acquisition phase, US-expectancy ratings, as well as anticipatory SCRs increased for the CS+1 and the CS+2 over trials while it decreased for the CS-. At the end of this phase, US-expectancy ratings, anticipatory SCRs, and negative valence and arousal ratings were all comparably high for the CS+1 and the CS+2, and significantly higher than they were for the CS-. These results indicate successful differential fear acquisition (see Fig. 2).

**Fig. 3:**
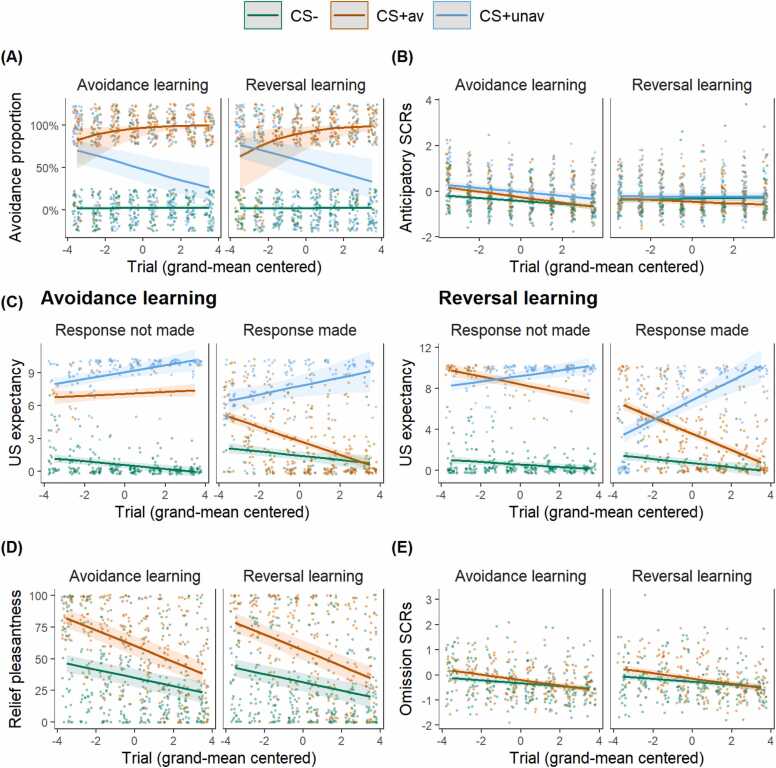
**Avoidance learning effects.** (A) The proportion of avoided trials; (B) Anticipatory SCR; (C) US expectancy in case an avoidance response was made and in case an avoidance response was not made; (D) Relief-pleasantness ratings. (E) US-omission-induced SCRs. CS+av = CS+ signaling avoidable US; CS+unav = CS+ signaling unavoidable US. Dots represent individual participant data; smooth lines represent the best model fit to the data; colored bands around the lines represent 95% confidence level.

With regards to the avoidance learning phases, the results were similar as in Leng et al.[14] (see Fig. 3). Participants showed increased avoidance responses towards the CS+av and reduced avoidance responses towards both the CS+unav and the CS- as the task progressed. On average, participants had high US expectancy towards the CS+unav but low US expectancy towards the CS-, regardless of avoidance responses. As expected, participants had lower US expectancy towards the CS+av if they had performed an avoidance response than if they had not. As for relief pleasantness, participants had higher ratings at US omissions following the CS+av compared to the CS- throughout the avoidance learning phases. As expected, relief pleasantness decreased as learning progressed and this decrease was steeper for the CS+av than for the CS-. A similar pattern was found for the omission-induced SCRs, but only during the reversal learning phase. Collectively, these results suggest that participants learned the association between the avoidance response and its contingent outcome as the task progressed.

### 4.3 Hypothesis 1a - Higher levels of anhedonia are associated with less relief pleasantness

**Fig. 4:**
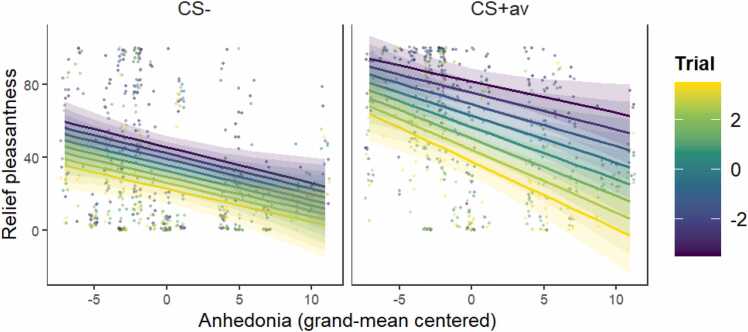
**Higher levels of anhedonia are associated with less relief pleasantness.** CS+av = the CS+ signaling avoidable US. The variable *Trial* was mean-centered with larger negative values representing earlier trials and larger positive values representing later trials. Dots represent individual participant data; smooth lines represent the best model fit to the data; colored bands around the lines represent 95% confidence level.

When SHAPS scores were added as a covariate of interest into the avoidance learning model for relief pleasantness, the final model included the three-way CS * Trial * SHAPS interaction. The results showed significant effects of CS, $\chi^{2}$(1) = 52.69*, p* < .001, Trial, $\chi^{2}$(1) = 55.51*, p* < .001, SHAPS, $\chi^{2}$(1) = 8.66, *p* < .001, and their three-way interaction, $\chi^{2}$(1) = 9.49, *p* < .001. Post-hoc comparisons revealed that participants with higher SHAPS scores had lower relief-pleasantness ratings for both CS+av (*β*= −2.49, *SE* = 0.66, 95% CI = [−3.83, −1.15]) and CS- (*β*= −1.77, *SE* = 0.79, 95% CI = [−3.37, −0.17]). While relief-pleasantness ratings decreased over trials for both CS+av (*β*= −6.20, *SE* = 0.50, 95% CI = [−7.21, −5.20]) and CS- (*β*= −3.24, *SE* = 0.48, 95% CI = [−4.19, −2.29]), the decrease for CS+av was faster for participants with higher SHAPS scores (difference in slopes: *β*= 2.96, *SE* = 0.48, *t*(1096.15) = 6.21, *p* < .001; see Fig. 4, the line gaps are larger from purple lines to yellow lines for higher anhedonia scores). All significant effects remained significant with other variables (age, gender, QIDS, STAI-T, IUS, DTS, general US unpleasantness) included as covariates, suggesting that these are relatively robust effects. Notably, no effect of these covariates was found (detailed results can be found in the Supplementary Material).

### 4.4 Hypothesis 1b - Higher levels of anhedonia are associated with lower omission-induced SCRs

No effect of SHAPS was found when added into the avoidance learning model of omission-induced SCR either before or after any covariate of no interest. Notably, participants with higher STAI-T scores showed significantly higher omission-induced SCRs (*β* = 0.01, 95% CI = [0.00, 0.02], *p* < .05). Detailed results can be found in the Supplementary Material.

### 4.5 Hypothesis 2 - Higher levels of anhedonia are associated with less avoidance

**Fig. 5:**
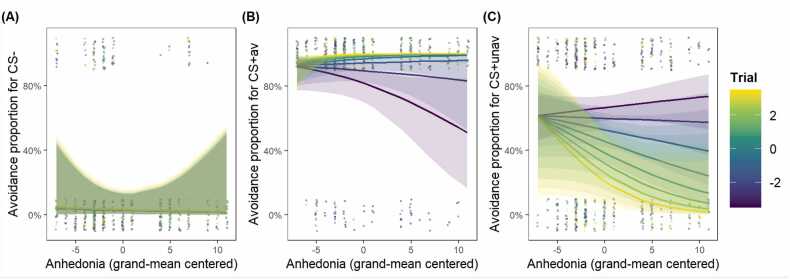
**Higher levels of anhedonia are associated with less avoidance for CS+av during early trials.** CS+av = the CS+ signaling avoidable US. CS+unav = CS+ signaling unavoidable US. The variable *Trial* was mean-centered with larger negative values representing earlier trials while larger positive values representing later trials. Dots represent individual participant data; smooth lines represent the best model fit to the data; colored bands around the lines represent 95% confidence level.

When SHAPS scores were added as a covariate of interest into the avoidance learning model for avoidance responses, the final model failed to meet the model assumptions. Therefore, we tested this hypothesis separately for each CS. The results showed that only for the CS+av, the Trial * SHAPS interaction was significant, where participants with higher SHAPS scores executed fewer avoidance responses during early trials (*Z* = 1.09, *p* < .001; see Fig. 5B). These results remained after the other covariates of no interest (age, gender, QIDS, STAI-T, IUS, DTS, general US unpleasantness) were added in. Again, no effect of these covariates was found (detailed results can be found in the Supplementary Material).

**Fig. 6:**
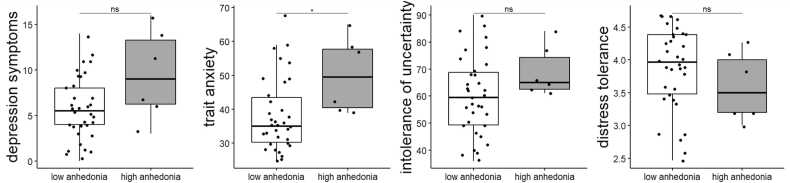
**Anhedonia-related difference in other trait measures.** Dots represent individual participant scores. Significant differences were tested with Welch’s *t*-test.

To further explore the behavioral profile of participants with high anhedonia scores, we separated the current sample based on a cut-off score for SHAPS [54] that categorizes participants into high (n = 6) or low (n = 34) anhedonia groups to allow a closer examination of the trial-by-trial trajectory for avoidance responses, relief pleasantness, and US expectancy ratings. Note that the high anhedonia group scored higher on trait anxiety (Fig. 6). However, with an extremely small sample size (n = 6) in the high anhedonia group, this result should be treated with caution.

**Fig. 7:**
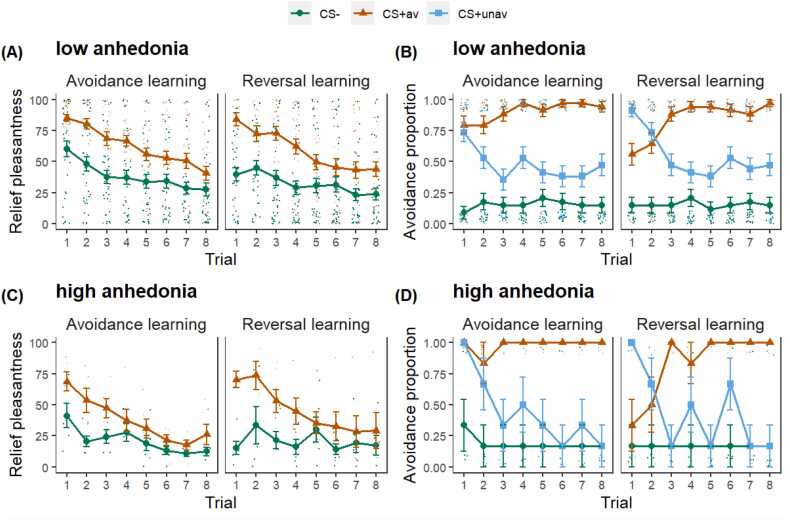
**Trial-by-trial data for relief pleasantness and avoidance responses.** (A & B) Relief pleasantness and avoidance proportion for participants without anhedonia; (C & D) Relief pleasantness and avoidance proportion for participants with anhedonia. Dots represent individual participant data. Error bars represent standard error of the mean.

When comparing relief pleasantness between low and high anhedonia groups (Figs. 7A and C), we noticed that the lower level of relief pleasantness reported by participants with low anhedonia was not merely a result of overall lower levels across the learning process, but also a lower initial relief level. When comparing the avoidance responses (Figs. 7B and D), the high anhedonia group rarely avoided on CS- trials and had a faster decrease in avoidance responses towards CS+unav. For the high anhedonia group, the effect found in support of Hypothesis 2 (fewer avoidance responses towards CS+av on early trials) seemed to be driven by lesser avoidance responses at the early stages of reversal learning, which is likely due to a larger drop in avoidance responses towards CS+unav during the avoidance learning phase.

### 4.3 Exploratory analyses

#### 4.3.1 More unexpected US-omission leads to more relief

We exploratorily examined the relationship between unexpected US omissions (indicated by both US-expectancy ratings and anticipatory SCRs) and relief experience afterward (indicated by both relief-pleasantness ratings and omission SCRs). Since these variables were registered on a trial-by-trial basis for each participant, repeated-measures correlation [55] was used to estimate the correlation coefficients to account for the non-independence of the data points and only capture the common intra-individual association. Results (see Table 2) showed that higher US-expectancy ratings were associated with higher anticipatory SCRs while higher relief-pleasantness ratings were associated with higher omission SCRs. Importantly, both US-expectancy ratings and anticipatory SCRs were positively associated with relief-pleasantness ratings and omission SCRs. Consistent with previous findings [14], when additionally adding the trial-by-trial US expectancy into the model where the effect of anhedonia on relief was tested, the previously observed anhedonia effect remained significant with the effect of US expectancy also being significant (Supplementary Material Table S9). These results further support the robustness of the observed anhedonia effect on self-reported relief pleasantness.

**Table 2:** Trial-by-trial repeated-measures correlations between measures.

		Anticipatory SCR		Relief pleasantness		Omission SCR	
US expectancy	All trials	0.17^***^	[0.11, 0.22]	0.61^***^	[0.58, 0.65]	0.23^***^	[0.16, 0.29]
	CS+av trials	0.11*	[0.02, 0.20]	0.61^***^	[0.56, 0.66]	0.32^***^	[0.23, 0.41]
	CS- trials	0.18^***^	[0.08, 0.27]	0.46^***^	[0.40, 0.52]	0.21^***^	[0.12, 0.30]
Anticipatory SCR	All trials		—	0.21^***^	[0.14, 0.27]	0.23^***^	[0.17, 0.30]
	CS+av trials		—	0.17^**^	[0.07, 0.26]	0.29^***^	[0.18, 0.40]
	CS- trials		—	0.12*	[0.02, 0.21]	0.25^***^	[0.13, 0.36]
Relief pleasantness	All trials		—		—	0.20^***^	[0.14, 0.27]
	CS+av trials		—		—	0.23^***^	[0.13, 0.32]
	CS- trials		—		—	0.21^***^	[0.11, 0.30]

Note. * *p* < .05, ** *p* < .01, *** *p* < .001. [95% CI], The sample size *N* = 28 for all SCR-related results while *N* = 40 for the rest.

#### 4.3.2 More relief pleasantness is not associated with more active avoidance

When adding the overall avoidance proportion as the outcome variable, and average relief pleasantness, phase, and their interaction term as predictors, no significant effect of relief pleasantness was found (Detailed results can be found in the Supplementary Material).

## 5 Discussion

Active avoidance is an adaptive coping strategy, but only when it is in line with the actual level of threat. Avoidance becomes maladaptive when it is out of proportion to the actual degree of threat - falling short is just as bad as going beyond. While excessive and persistent avoidance are well-recognized characteristics in anxiety-related disorders, insufficient threat avoidance is less understood. A large-scale online study gathered initial evidence for anhedonia as a potential factor contributing to insufficient threat avoidance [14], but the validity of the online task as a measure of active threat avoidance remained unclear. The purpose of the current study was to examine the influence of anhedonia on active avoidance in a well-validated fear-learning paradigm. A successful conceptual replication would lend support to our theory that anhedonia decreases relief and adaptive avoidance, which might culminate into learned helplessness as in the case of depression.

Consistent with our previous study [14], at the level of self-report, participants who scored higher for anhedonia experienced weaker relief pleasantness during threat omissions – including both passive threat omissions (no action was engaged) following the CS- trials and active threat omissions (after active avoidance actions) following the CS+av trials. Behaviorally, at the early stages of each phase, participants who scored higher for anhedonia executed fewer avoidance actions, specifically for the CS+av trials. Thus, at first sight, the results seem to be in line with the anhedonia-relief theory and our hypothesis. However, when zooming into the trial-by-trial development for both relief pleasantness and avoidance responses, the picture changes, as we discuss below.

**Fig. 8:**
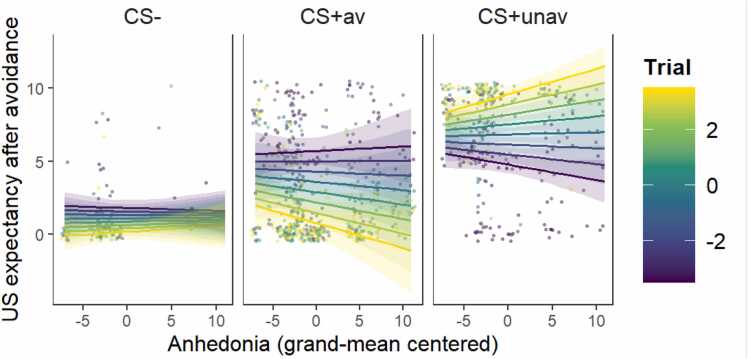
**US expectancy for trials with avoidance.** After avoidance, participants with higher anhedonia showed faster decreasing US expectancy for the CS+av trials and faster increasing US expectancy for the CS+unav trials. CS+av = the CS+ signaling avoidable US; CS+unav = the CS+ signaling unavoidable US. The variable *Trial* was mean-centered with larger negative values representing earlier trials while larger positive values representing later trials. Dots represent raw data from each participant; smooth lines represent the best model fit to the data; colored bands around the lines represent 95% confidence level.

The current study employed a learning task. As learning progressed, the association between successful avoidance response and its subsequent threat omission was gradually anticipated and therefore, relief pleasantness induced by threat omissions would diminish. At the same time, the association between ineffective avoidance response and its ensuing failure in preventing threats from occurring should also be gradually acquired. In this discriminated avoidance learning task, a better learner is expected to demonstrate increased successful avoidance but decreased ineffective avoidance (comparing Figs. 7B and D). Additionally, a better learner should have decreased US expectancy for CS+av trials but increased US expectancy for CS+unav trials after an avoidance response was made (Fig. 8 shows the results when adding SHPAS into the avoidance learning model with US expectancy being the outcome variable). Taken together, participants who scored higher for anhedonia in the current study seem to be learning more aptly, which complicates the interpretation of the current results.

Although we found that participants who scored higher for anhedonia experienced a faster decline of relief pleasantness as the task progressed, we are unable to tease apart whether this faster decline is due to greater anhedonia directly or merely reflective of a faster learning curve. Yet, participants who scored higher for anhedonia did report lower relief pleasantness at the beginning of the avoidance learning phase (where no learning had yet occurred and relief pleasantness at threat omissions was supposed to be high) as well as significantly lower relief pleasantness as retrospectively measured at the end of the experiment. When all the evidence is taken into account, it does seem that the presence of anhedonia did attenuate the relief experience induced by active threat omissions.

In terms of the avoidance responses, we found that participants who scored higher for anhedonia executed fewer avoidance responses towards the CS+av, specifically at the early stages of the reversal learning phase. A closer examination of Fig. 7D reveals that this was probably caused by a larger drop of ineffective avoidance towards the CS+unav in the preceding phase compared with participants scoring lower for anhedonia. As a result, participants with high anhedonia needed more trials to learn that the CS+unav had effectively become the CS+av. In fact, they had a larger drop for the ineffective avoidance towards the CS+unav in both phases, which could be further evidence of better learning in these participants. Of note, the better avoidance learning, if present, cannot be attributed to a better fear acquisition as there was no effect of anhedonia on fear acquisition as indicated by US expectancy. Particularly, participants who scored higher for anhedonia also exhibited less unnecessary avoidance towards the CS- in the current study.

With regard to skin conductance, no anhedonia effect was observed. Notably, we did find anhedonia effect on relief pleasantness ratings as well as positive correlations between omission SCR and relief pleasantness ratings for both the CS+av and the CS- trials. Given these findings, the null effect of anhedonia on SCR might due to the fact that the sample size for testing this hypothesis was smaller (*N* = 28 vs. *N* = 40), therefore, not enough power to detect the anhedonia effect. Instead, we found that participants with higher trait anxiety in the current study showed higher threat-omission-induced skin conductance responses, which is consistent with previous findings that anxious people tend to experience stronger relief at threat omissions [9], [10]. Importantly, we also found that trial-by-trial relief pleasantness was positively correlated with trial-by-trial threat-omission-induced SCRs, which further supports that the arousal at threat omissions is positive.

With different anhedonia questionnaires and different types of threats (aversive pictures versus electrical stimulus), both the previous study [14] and the current one have confirmed that higher levels of anhedonia are associated with lower levels of relief. However, when comparing the findings from the two studies, we found that the impairing effect of anhedonia on relief seems to be a function of relief strength. For instance, in the previous study [14], anhedonia had a larger effect on relief pleasantness during early (when relief pleasantness was relatively higher in that study) compared to late CS+av trials in the avoidance learning phase (although not statistically significant). In contrast, anhedonia in the current study had its largest effect on relief during late rather than early trials for CS+av, when relief pleasantness was relatively lower. In addition, the predictive effect of US unpleasantness overshadowed the effects of anhedonia on relief in the previous study [14], whereas this was not the case in the current study. It is plausible that these different boundary conditions for the anhedonia effect are rooted in the overall US aversiveness - the much more aversive US used in the current study led to higher relief pleasantness overall. Although the relief pleasantness decreased in both studies over the course of learning, the relief pleasantness towards CS+av at the end of the task (i.e., at its lowest) in the current study might still have been higher than the initial (i.e., highest) relief pleasantness towards CS+av in the previous study [14]. The observed anhedonia effects suggest that an impairing effect of anhedonia might only be detected when relief pleasantness is at a certain level – not too strong nor too weak.

Remarkably, all these anhedonia-related effects remained when accounting for other factors (age, gender, QIDS, STAI-T, IUS, DTS, US unpleasantness). Especially for the relief pleasantness, the effect of anhedonia was significant beyond US unpleasantness and US expectancy, which suggests that threat-omission-induced relief pleasantness is not purely a function of US aversiveness and is more than just a surprise effect (an unsigned prediction error). Rather, it seems specific to the *rewarding value* of threat omissions. Additionally, the anhedonia effect was observed over and above general depressive symptoms (sleep disturbance, sad mood, fatigue, etc.), suggesting the distinct role of anhedonia in the processing of threat-omission-induced relief. While active threat avoidance has been investigated mainly for anxiety-related disorders with the focus of disconfirming irrational threat beliefs [6], the current findings highlight the need for more attention to potential positive affect impairment that may impede treatment [31]. Moreover, anhedonia has mainly been researched in understanding deficient in pleasure cycle and positive affectivity [56]. Based on the current findings, active threat avoidance might engage both negative and positive system, which anhedonia might play a role. Therefore, broadening the scope of anhedonia affect beyond positive experiences could help us gain better insights in understanding anhedonia.

The current study has several limitations. First of all, the learning task itself induced a dynamic decrease in relief pleasantness over trials, making it difficult to draw definite conclusions regarding the effects of anhedonia. That is, the necessary conditions to observe an effect of anhedonia on relief are still unclear. Future studies could employ tasks where relief levels can remain relatively high or systematically manipulated to closely examine whether anhedonia has its effect only at a certain range. Secondly, the avoidance action in the current study was simply a button press (i.e., a binary measure), leaving limited room to explore individual differences in terms of to what extent participants wanted to avoid. Future studies could consider more sensitive measures of avoidance tendencies [57], [58]. Thirdly, anhedonia scores in the current study were not normally distributed, more definite findings can be derived from a larger sample size with more variation in anhedonia measurement in the future. Alternatively, comparing selected samples with and without anhedonia can also inform us of a clearer anhedonia effect. Finally, it is worth noting that the majority of the current sample was female, thus caution should be taken when generalizing the current findings in male population.

## 6 Conclusions

To conclude, the current study further confirmed that the presence of anhedonia could cause insufficient active threat avoidance and impair the rewarding value of relief at successful threat omissions. Although self-reported relief pleasantness was highly correlated with corresponding skin conductance responses elicited by omissions, anhedonia-related impairment was only observed in self-reported pleasantness of relief but not the psychophysical responses elicited by relief. Importantly, the impairing effect of anhedonia on relief seems to be a function of relief strength, which calls for future investigation.

## Declaration of Competing Interest

None.
