# Supplementary material for: Anhedonia influences threat avoidance and relief: A conceptual replication
Source: J Mood Anxiety Disord. 2024 Jan 17;5:100050. doi: 10.1016/j.xjmad.2024.100050 (PMC12244109; doi:10.1016/j.xjmad.2024.100050)
Supplement: Supplementary file 1 — Supplementary material. [file mmc1.docx]

Anhedonia influences threat avoidance and relief: a conceptual replication

**Supplementary Material**

Lu Leng, Tom Beckers, and Bram Vervliet

# Scatter plots for all the variables reported in Table 1


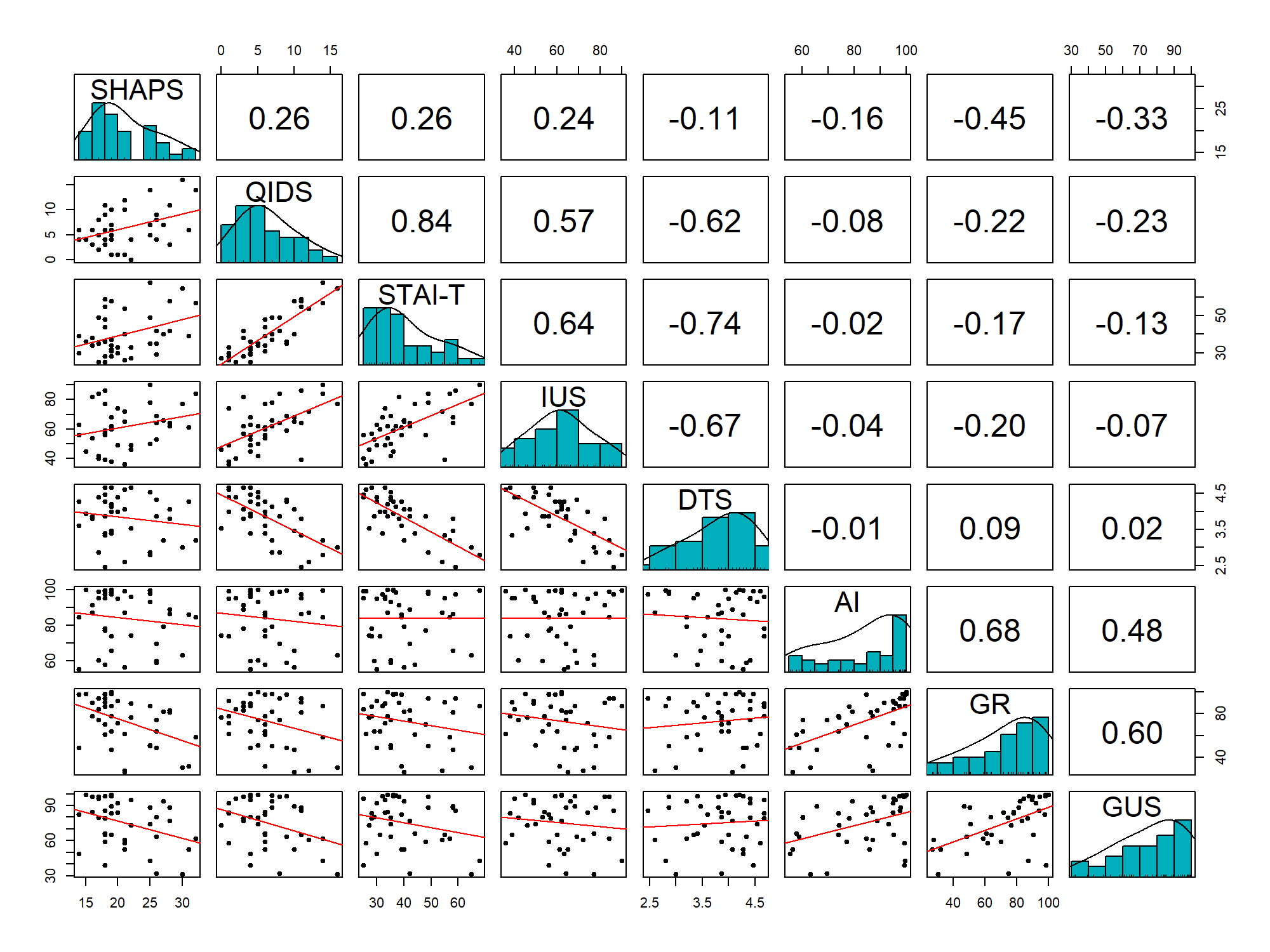


**Figure S1. Scatter plots for all variables reported in Table 1.** *Note*. SHAPS = The Snaith Hamilton Pleasure Scale; QIDS = Quick Inventory of Depressive Symptoms; STAI-T = The State and Trait Anxiety Inventory - Trait Version; IUS = Intolerance of Uncertainty Scale; DTS = Distress Tolerance Scale; AI = avoidance intention; GR = general relief pleasantness; GUS = general US unpleasantness.

# Statistical analyses

All the models were built using the ***lme4*** package (Bates et al., 2014) and significance was calculated using the ***lmerTest*** package (Kuznetsova et al., 2017), following the relevant model specification and reporting guidelines (Bates et al., 2018; Brown, 2021; Luo et al., 2021). Unrestricted maximum likelihood (ML) was used for all models to control the optimization procedure for the parameter estimates. All categorical predictors (e.g., Phase, CS) were coded using sum to zero contrasts while continuous predictors (e.g., Trial) were grand-mean centered to make interpretations easier and to reduce multicollinearity when an interaction term was included (Bickel, 2007). A random intercept over all participants was included in all models to account for the repeated-measures nature of the data. Random slopes were tested and kept if they significantly improved model fit and achieved model convergence. The model with the best goodness-of-fit was selected based on the likelihood-ratio test. Once the best model was selected, the significance of fixed effects was evaluated by the likelihood-ratio test. The ***tab_model*** fuction was used to provide summaries of standardized coefficients, which provide information about the magnitude of the effect (Ferron et al., 2008). The marginal means were estimated and post-hoc comparisons were conducted for fixed effects using ***emmeans*** and ***emtrends*** packages with Bonferroni correction and the Satterthwaite approximation (Russell, 2021). The nature of any significant interaction effect was additionally inspected with the interaction plot. The fixed effects of other covariates of no interest (age, gender, US unpleasantness, QIDS, STAI-T, IUS, DTS) were added into the final models (both separately and simultaneously) to check the robustness of our findings. Model assumptions were visually checked with the output from ***check_model*** function. When in doubt, extra tests were run from the ***DHARMa*** package (Hartig, 2022). Predictors with variance inflation factor (VIF) bigger than 5 were removed from the model (Daoud, 2017). Except for Bonferroni-corrected estimates, the significance threshold was set at *p* = .05.

# Model summaries

## Fear acquisition

**US expectancy.** The final model included the fixed effects of CS, Trial, and their interaction with random slopes for CS. Results showed all the fixed effects were significant, CS: $\chi^{2}$(2) = 109.42, *p* < .001; Trial: $\chi^{2}$(1) = 116.91, *p* < .001; CS * Trial: $\chi^{2}$(2) = 285.83, *p* < .001, see Figure 2A. Post-hoc pairwise comparisons showed that on average, the ratings between the CS+1 (*M* = 7.48, *SE* = 0.18) and the CS+2 (*M* = 7.81, *SE* = 0.16) were comparably high, $\beta$ =-0.32, *SE* = 0.21, *t*(40.27) = -1.56, *p* = .383, which were both higher than the ratings to the CS- (*M* = 1.55, *SE* = 0.17, compared to the CS+1: $\beta$ =-5.93, *SE* = 0.3, *t*(39.83) = -19.98, *p* < .001; compared to the CS+2: $\beta$ =-6.25, *SE* = 0.26, *t*(40.16) = -23.95, *p* < .001). While the US-expectancy ratings increased for the CS+1 ($\beta$ = 1.59, 95% CI [1.39, 1.79]) and CS+2 ($\beta$ = 1.48, 95% CI [1.29, 1.68]) comparably over trials, difference in slopes: $\beta$ =0.11, *SE* = 0.14, *t*(351.11) = 0.75, *p* = 1.000, the US-expectancy ratings decreased for the CS- ($\beta$ = -1.03, 95% CI [-1.22, -0.83]). Notably, at the end of the acquisition phase, US expectancy were significantly lower for the CS- (*M* = 0.04, *SE* = 0.23) compared to the CS+1 (*M* = 9.83, *SE* = 0.24, $\beta$ =-9.79, *SE* = 0.36, *t*(86.62) = -26.95, *p* < .001) and CS+2 (*M* = 10.00, *SE* = 0.22, $\beta$ =-9.95, *SE* = 0.33, *t*(103.06) = -29.76, *p* < .001).

**Anticipatory SCR.** The final model included the fixed effects of CS, Trial, and their interaction. Results showed significant effects of CS: $\chi^{2}$(2) = 39.64, *p* < .001 and CS * Trial interaction: $\chi^{2}$(2) = 24.06, *p* < .001, see Figure 2B. Post-hoc pairwise comparisons showed that on average, the SCRs between the CS+1 (*M* = -0.06, *SE* = 0.06) and the CS+2 (*M* = -0.25, *SE* = 0.06) were comparably high, $\beta$ =0.19, *SE* = 0.09, *t*(308) = 2.18, *p* = .091, which were both higher than the SCR to the CS- (*M* = -0.61, *SE* = 0.06, compared to the CS+1: $\beta$ =-0.56, *SE* = 0.09, *t*(308) = -6.40, *p* < .001; compared to the CS+2: $\beta$ =-0.37, *SE* = 0.09, *t*(308) = -4.22, *p* < .001). While the anticipatory SCR increased for the CS+1 ($\beta$ = 0.19, 95% CI [0.08, 0.29]) and CS+2 ($\beta$ = 0.11, 95% CI [0.00, 0.21]) comparably over trials, difference in slopes: $\beta$ = 0.08, *SE* = 0.08, *t*(308) = 1.01, *p* = .940), the SCRs decreased for the CS- ($\beta$ = -0.19, 95% CI [-0.29, -0.08]). Notably, at the end of the acquisition phase, SCRs were significantly lower for the CS- (*M* = -0.89, *SE* = 0.11) compared to the CS+1 (*M* = 0.22, *SE* = 0.11, $\beta$ = -1.11, *SE* = 0.15, *t*(308) = -7.63, *p* < .001) and CS+2 (*M* = -0.09, *SE* = 0.11, $\beta$ =-0.81, *SE* = 0.15, *t*(308) = -5.52, *p* < .001).

**CS valence and arousal.** RM-ANOVAs and post-hoc comparisons revealed that valence and arousal for all CSs were comparable before the fear acquisition phase. After the fear acquisition phase, compared to the CS- (valence: *M* = 19.00, *SD* = 22.2; arousal: *M* = 19.30, *SD* = 19.20), both the CS+1 (valence: *M* = 81.50, *SD* = 21.90; arousal: *M* = 76.70, *SD* = 23.20) and the CS+2 (valence: *M* = 84.00, *SD* = 15.80; arousal: *M* = 72.50, *SD* = 26.20) were rated more unpleasant (CS+1: *t*(39) = 10.66, *p* < .001, *d* = 2.83; CS+2: *t*(39) = 11.69, *p* < .001, *d* = 3.37) as well as more stimulating (CS+1: *t*(39) = 9.79, *p* < .001, *d* = 2.69; CS+2: *t*(39) = 8.82, *p* < .001, *d* = 2.32, see Fig. 2C and 2D).

## Avoidance learning

**Avoidance proportion.** The final model included the fixed effects of Phase, CS, Trial, and two interaction terms (Phase * CS, CS * Trial) with random slopes for CS. Results showed significant effects of CS: $\chi^{2}$(2) = 75.95*, p* <.001, Phase * CS: $\chi^{2}$(2) = 14.91, *p* <.001, CS * Trial: $\chi^{2}$(2) = 131.47, *p* <.001. Post-hoc pairwise comparisons showed that averagely, the proportion of avoided trials decreased from the CS+av over the CS+unav to the CS- in both phases (*p*s < .001). The proportion of avoided CS+av trials was higher in the avoidance learning phase compared to the reversal learning phase (*Z* = 3.33, *p* < .001), while the avoided trials were comparable for the CS+unav and for the CS- in both phases. In both learning phases, while avoidance towards the CS+av increased over trials (*β* = 0.55, 95% CI [0.39, 0.71]), it decreased for CS+unav (*β* = -0.27, 95% CI [-0.35, -0.18]) and remained low for the CS- (*β* = 0.03, 95% CI [-0.13, 0.20]). See Figure 3A for an overview of the effects.

**US expectancy.** The final model included the fixed effects of Phase, CS, Trial, Avoided, and the four-way interaction term with random slopes for CS and Avoided. Results showed significant interaction effect of Phase * CS * Trial * Avoided: $\chi^{2}$(2) = 8.39*, p* < .001. Post-hoc pairwise comparisons revealed that there was no difference in US-expectancy ratings between Response made (*M* = 1.76, *SE* = 0.36 in avoidance learning; *M* = 1.25, *SE* = 0.37 in reversal learning) and Response not made (*M* = 0.54, *SE* = 0.13 in avoidance learning; *M* = 0.50, *SE* = 0.13 in reversal learning) for the CS- trials in both phases (*p* > .05). In contrast, US-expectancy ratings were higher for Response made compared to Response not made for both CS+unav and CS+av trials (*p*s < .001). Regardless of the avoidance responses, average US-expectancy ratings were decreasing from CS+unav (*M* = 8.40, *SE* = 0.19 in the avoidance learning; *M* = 7.85, *SE* = 0.20 in the reversal learning) to CS+av (*M* = 4.67, *SE* = 0.32 in the avoidance learning; M = *M* = 5.78, *SE* = 0.29 in the reversal learning) to CS- in both phases (*M* = 1.15, *SE* = 0.20 in the avoidance learning; *M* = 0.87, *SE* = 0.20 in the reversal learning; *p*s < .001). In both phases, regardless of the avoidance actions, US-expectancy ratings increased for the CS+unav and decreased for the CS- over the trials. In contrast, US-expectancy ratings remained high for Response not made CS+av trials during the avoidance learning phase, but decreased for the Response not made CS+av trials during the reversal learning phase. For Response made CS+av trials in both phase, US-expectancy ratings decreased over the trials. See Figure 3C for an overview of the effects.

**Anticipatory SCR.** The final model included the fixed effects of Phase, CS, Trial, Avoided, and two-way interaction terms (Phase * CS, Trial * Phase, CS* Trial, CS * Avoided_factor) with random slopes for CS. Results showed significant effect of CS: $\chi^{2}$(2) = 17.32 *, p* < .001; Trial: $\chi^{2}$(1) = 32.35 *, p* < .001; Avoided: $\chi^{2}$(1) = 9.04 , *p* < .001; Phase * CS: $\chi^{2}$(2) = 14.45 *, p* < .001; Phase * Trial: $\chi^{2}$(1) = 24.27*, p* < .001; CS * Trial: $\chi^{2}$(2) = 3.34, *p* < .001. Post-hoc pairwise comparisons revealed that in both phases, regardless of the avoidance response, anticipatory SCRs were comparably high for the CS+unav and CS+av (*p*s > .05), which were both higher than CS- (*p*s < .05). See Figure 3B for an overview of the effects.

**Relief pleasantness.** The final model included the fixed effects of Phase, CS, Trial, and CS * Trial interaction with random slopes for CS and Trial. Results showed that the fixed effect of CS, $\chi^{2}$(1) = 51.67*, p* < .001, Trial, $\chi^{2}$(1) = 55.31*, p* < .001, Phase, $\chi^{2}$(1) = 10.15, *p* < .001, and CS * Trial: $\chi^{2}$(1) = 37.19*, p* < .001. Post-hoc comparisons revealed that on average in both phases, relief-pleasantness ratings for the CS- (*M* = 34.56, *SE* = 3.97 in the avoidance learning; *M* = 31.13, *SE* = 3.97 in the reversal learning) were significantly lower compared to the CS+av (*M* = 59.37, *SE* = 3.67 in the avoidance learning; *M* = 55.95, *SE* = 3.68 in the reversal learning, *p*s < .001. In both phases, while the relief-pleasantness decreased for both the CS- ($\beta$ = -3.24, 95% CI [-4.19, -2.28]) and the CS+av ($\beta$ = -6.2, 95% CI [-7.21, -5.19]), the decrease was faster for the CS+av than the CS- (difference in slope: $\beta$ = 2.96, *SE* = 0.48, *t*(1096.01) = 6.15, *p* < .001). See Figure 3D for an overview of the effects.

**Omission SCR.** The final model included the fixed effects of Phase, CS, Trial, and CS * Trial interaction with random slopes for Phase. Results showed that the fixed effect of CS, $\chi^{2}$(1) = 7.80, *p* < .001, Trial, $\chi^{2}$(1) = 65.16*, p* < .001, and CS * Trial: $\chi^{2}$(1) = 5.26*, p* < .001. Post-hoc comparisons revealed that the average omission-induced SCRs were higher for the CS+av (*M* = -0.19, *SE* = 0.05) compared to the CS- (*M* = -0.31, *SE* = 0.05). While omission-induced SCRs decreased for both the CS+av and the CS- in both phases, this decrease was faster for the CS+av compared to the CS- (difference in slope: $\beta$ = 0.05, *SE* = 0.02, *t*(793.79) = 2.30, *p* < .05). See Figure 3E for an overview of the effects.

# Model results of hypothesis testing

## Hypothesis 1

- - 1. Hypothesis 1a

In the following tables (Table S1-3), each column represents one model with the outcome variable always being the relief pleasantness. The first column represents the model for testing hypothesis 1a without any covariate being added. The column names on top represents the covariate that was added into the hypothesis testing model, and its effect corresponds to the effect of the predictor ‘covariate’*.* Notably, the original three-way interaction remains significant in all the models and only the US unpleasantness was found to be associated with relief pleasantness (Table S1). To follow up the three-way interaction, the original model for hypothesis testing was run for each CS, separately. The sensitivity check was also done with these models and the results can be found in the following two tables (Table S2-3).

# Table S1

*Model summaries for Hypothesis 1a*

|  |  | | **USunpleasantness_c** | | **Age_c** | | **Gender** | | **QIDS_c** | | **STAI_c** | | **IUS_c** | | **DTS_c** | |
| --- | --- | --- | --- | --- | --- | --- | --- | --- | --- | --- | --- | --- | --- | --- | --- | --- |
| *Predictors* | *Estimates* | *CI* | *Estimates* | *CI* | *Estimates* | *CI* | *Estimates* | *CI* | *Estimates* | *CI* | *Estimates* | *CI* | *Estimates* | *CI* | *Estimates* | *CI* |
| (Intercept) | 45.87 ^***^ | 39.61 – 52.14 | 45.86 ^***^ | 40.33 – 51.39 | 45.90 ^***^ | 39.97 – 51.83 | 44.69 ^***^ | 36.36 – 53.01 | 45.88 ^***^ | 39.69 – 52.06 | 45.87 ^***^ | 39.68 – 52.07 | 45.87 ^***^ | 39.61 – 52.14 | 45.87 ^***^ | 39.63 – 52.11 |
| Phase1 | 1.69 ^**^ | 0.64 – 2.73 | 1.69 ^**^ | 0.65 – 2.74 | 1.69 ^**^ | 0.65 – 2.74 | 1.55 ^**^ | 0.48 – 2.61 | 1.69 ^**^ | 0.64 – 2.73 | 1.69 ^**^ | 0.64 – 2.73 | 1.69 ^**^ | 0.64 – 2.73 | 1.69 ^**^ | 0.64 – 2.73 |
| SHAPS c | -2.12 ^**^ | -3.45 – -0.78 | -1.39 ^*^ | -2.64 – -0.15 | -2.39 ^***^ | -3.69 – -1.09 | -1.94 ^*^ | -3.46 – -0.43 | -2.28 ^**^ | -3.69 – -0.86 | -2.32 ^**^ | -3.72 – -0.92 | -2.15 ^**^ | -3.53 – -0.78 | -2.18 ^**^ | -3.52 – -0.83 |
| CS1 | -12.67 ^***^ | -15.03 – -10.31 | -12.66 ^***^ | -15.02 – -10.30 | -12.70 ^***^ | -15.07 – -10.32 | -12.80 ^***^ | -15.21 – -10.38 | -12.68 ^***^ | -15.04 – -10.31 | -12.67 ^***^ | -15.03 – -10.31 | -12.67 ^***^ | -15.03 – -10.31 | -12.67 ^***^ | -15.03 – -10.31 |
| Trial c | -4.72 ^***^ | -5.56 – -3.88 | -4.71 ^***^ | -5.54 – -3.87 | -4.72 ^***^ | -5.56 – -3.88 | -4.76 ^***^ | -5.62 – -3.90 | -4.72 ^***^ | -5.56 – -3.88 | -4.72 ^***^ | -5.56 – -3.88 | -4.72 ^***^ | -5.56 – -3.88 | -4.72 ^***^ | -5.56 – -3.88 |
| Phase1 × SHAPS c | -0.25 ^*^ | -0.48 – -0.03 | -0.25 ^*^ | -0.47 – -0.03 | -0.26 ^*^ | -0.48 – -0.03 | -0.31 ^**^ | -0.55 – -0.08 | -0.25 ^*^ | -0.48 – -0.03 | -0.25 ^*^ | -0.48 – -0.03 | -0.25 ^*^ | -0.48 – -0.03 | -0.25 ^*^ | -0.48 – -0.03 |
| CS1 × Trial c | 1.48 ^***^ | 1.01 – 1.95 | 1.47 ^***^ | 1.00 – 1.94 | 1.48 ^***^ | 1.01 – 1.95 | 1.51 ^***^ | 1.03 – 1.99 | 1.48 ^***^ | 1.01 – 1.95 | 1.48 ^***^ | 1.01 – 1.95 | 1.48 ^***^ | 1.01 – 1.95 | 1.48 ^***^ | 1.01 – 1.95 |
| SHAPS c × CS1 | 0.34 | -0.16 – 0.84 | 0.35 | -0.15 – 0.85 | 0.34 | -0.16 – 0.85 | 0.29 | -0.24 – 0.82 | 0.34 | -0.16 – 0.85 | 0.34 | -0.16 – 0.84 | 0.34 | -0.16 – 0.84 | 0.34 | -0.16 – 0.84 |
| SHAPS c × Trial c | -0.11 | -0.29 – 0.07 | -0.11 | -0.28 – 0.07 | -0.11 | -0.29 – 0.07 | -0.13 | -0.31 – 0.06 | -0.11 | -0.29 – 0.07 | -0.11 | -0.29 – 0.07 | -0.11 | -0.29 – 0.07 | -0.11 | -0.29 – 0.07 |
| (SHAPS c × CS1) × Trial c | 0.16 ^**^ | 0.06 – 0.26 | 0.15 ^**^ | 0.06 – 0.25 | 0.16 ^**^ | 0.06 – 0.26 | 0.17 ^**^ | 0.06 – 0.27 | 0.16 ^**^ | 0.06 – 0.26 | 0.16 ^**^ | 0.06 – 0.26 | 0.16 ^**^ | 0.06 – 0.26 | 0.16 ^**^ | 0.06 – 0.26 |
| covariate |  |  | 0.49 ^***^ | 0.22 – 0.77 | -1.64 | -3.43 – 0.16 |  |  | 0.51 | -1.11 – 2.13 | 0.23 | -0.31 – 0.78 | 0.05 | -0.38 – 0.48 | -2.99 | -12.77 – 6.79 |
| covariate1 |  |  |  |  |  |  | 2.09 | -6.18 – 10.36 |  |  |  |  |  |  |  |  |
| **Random Effects** | | | | | | | | | | | | | | | | |
| σ^2^ | 337.50 | | 337.72 | | 337.37 | | 343.43 | | 337.46 | | 337.46 | | 337.49 | | 337.47 | |
| τ_00_ | 396.03 _PPN_ | | 305.39 _PPN_ | | 353.55 _PPN_ | | 415.84 _PPN_ | | 385.40 _PPN_ | | 386.65 _PPN_ | | 395.65 _PPN_ | | 392.93 _PPN_ | |
| τ_11_ | 46.02 _PPN.CS1_ | | 45.90 _PPN.CS1_ | | 46.54 _PPN.CS1_ | | 46.92 _PPN.CS1_ | | 46.14 _PPN.CS1_ | | 46.09 _PPN.CS1_ | | 46.02 _PPN.CS1_ | | 46.08 _PPN.CS1_ | |
|  | 5.03 _PPN.Trial_c_ | | 4.94 _PPN.Trial_c_ | | 5.05 _PPN.Trial_c_ | | 5.15 _PPN.Trial_c_ | | 5.05 _PPN.Trial_c_ | | 5.05 _PPN.Trial_c_ | | 5.04 _PPN.Trial_c_ | | 5.04 _PPN.Trial_c_ | |
| ρ_01_ | 0.31 | | 0.35 | | 0.29 | | 0.34 | | 0.30 | | 0.31 | | 0.31 | | 0.32 | |
|  | 0.36 | | 0.34 | | 0.28 | | 0.39 | | 0.33 | | 0.34 | | 0.35 | | 0.35 | |
| ICC | 0.59 | | 0.53 | | 0.56 | | 0.59 | | 0.58 | | 0.58 | | 0.59 | | 0.58 | |
| N | 40 _PPN_ | | 40 _PPN_ | | 40 _PPN_ | | 39 _PPN_ | | 40 _PPN_ | | 40 _PPN_ | | 40 _PPN_ | | 40 _PPN_ | |
| Observations | 1200 | | 1200 | | 1200 | | 1170 | | 1200 | | 1200 | | 1200 | | 1200 | |
| Marginal R^2^ / Conditional R^2^ | 0.315 / 0.716 | | 0.387 / 0.713 | | 0.340 / 0.711 | | 0.306 / 0.717 | | 0.320 / 0.714 | | 0.321 / 0.715 | | 0.315 / 0.716 | | 0.318 / 0.716 | |
| AIC | 10677.275 | | 10671.465 | | 10674.915 | | 10428.352 | | 10677.475 | | 10679.344 | | 10680.403 | | 10673.875 | |
| ** p<0.05   ** p<0.01   *** p<0.001* | | | | | | | | | | | | | | | | |

# Table S2

*Model summaries for Hypothesis 1a – CS+av*

|  |  | | **USunpleasantness_c** | | **Age_c** | | **Gender** | | **QIDS_c** | | **STAI_c** | | **IUS_c** | | **DTS_c** | |
| --- | --- | --- | --- | --- | --- | --- | --- | --- | --- | --- | --- | --- | --- | --- | --- | --- |
| *Predictors* | *Estimates* | *CI* | *Estimates* | *CI* | *Estimates* | *CI* | *Estimates* | *CI* | *Estimates* | *CI* | *Estimates* | *CI* | *Estimates* | *CI* | *Estimates* | *CI* |
| (Intercept) | 58.91 ^***^ | 52.80 – 65.02 | 58.81 ^***^ | 53.49 – 64.12 | 58.96 ^***^ | 53.14 – 64.79 | 55.82 ^***^ | 47.83 – 63.80 | 58.91 ^***^ | 52.79 – 65.02 | 58.91 ^***^ | 52.85 – 64.97 | 58.90 ^***^ | 52.75 – 65.06 | 58.91 ^***^ | 52.82 – 64.99 |
| Phase1 | 0.70 | -0.80 – 2.19 | 0.72 | -0.78 – 2.21 | 0.71 | -0.78 – 2.20 | 0.67 | -0.85 – 2.20 | 0.70 | -0.79 – 2.19 | 0.70 | -0.80 – 2.19 | 0.70 | -0.79 – 2.20 | 0.70 | -0.79 – 2.19 |
| Trial c | -6.13 ^***^ | -7.35 – -4.91 | -6.10 ^***^ | -7.30 – -4.89 | -6.14 ^***^ | -7.37 – -4.92 | -6.18 ^***^ | -7.43 – -4.93 | -6.13 ^***^ | -7.35 – -4.91 | -6.13 ^***^ | -7.36 – -4.91 | -6.12 ^***^ | -7.34 – -4.90 | -6.13 ^***^ | -7.35 – -4.91 |
| SHAPS c | -2.55 ^***^ | -3.85 – -1.24 | -1.87 ^**^ | -3.07 – -0.68 | -2.80 ^***^ | -4.08 – -1.53 | -2.15 ^**^ | -3.62 – -0.68 | -2.53 ^***^ | -3.93 – -1.14 | -2.65 ^***^ | -4.02 – -1.28 | -2.48 ^***^ | -3.84 – -1.13 | -2.58 ^***^ | -3.89 – -1.26 |
| Trial c × SHAPS c | -0.27 ^*^ | -0.53 – -0.01 | -0.26 ^*^ | -0.52 – -0.01 | -0.28 ^*^ | -0.54 – -0.02 | -0.30 ^*^ | -0.58 – -0.03 | -0.27 ^*^ | -0.53 – -0.01 | -0.27 ^*^ | -0.53 – -0.01 | -0.27 ^*^ | -0.53 – -0.01 | -0.27 ^*^ | -0.53 – -0.01 |
| covariate |  |  | 0.46 ^***^ | 0.20 – 0.73 | -1.52 | -3.25 – 0.20 |  |  | -0.04 | -1.60 – 1.52 | 0.12 | -0.40 – 0.64 | -0.08 | -0.49 – 0.33 | -1.46 | -10.82 – 7.91 |
| covariate1 |  |  |  |  |  |  | 5.28 | -2.48 – 13.03 |  |  |  |  |  |  |  |  |
| **Random Effects** | | | | | | | | | | | | | | | | |
| σ^2^ | 317.08 | | 318.39 | | 316.85 | | 323.20 | | 317.10 | | 316.99 | | 317.16 | | 317.04 | |
| τ_00_ | 360.40 _PPN_ | | 266.41 _PPN_ | | 325.09 _PPN_ | | 376.42 _PPN_ | | 361.18 _PPN_ | | 353.66 _PPN_ | | 365.71 _PPN_ | | 356.93 _PPN_ | |
| τ_11_ | 10.44 _PPN.Trial_c_ | | 10.03 _PPN.Trial_c_ | | 10.51 _PPN.Trial_c_ | | 10.66 _PPN.Trial_c_ | | 10.44 _PPN.Trial_c_ | | 10.49 _PPN.Trial_c_ | | 10.40 _PPN.Trial_c_ | | 10.47 _PPN.Trial_c_ | |
| ρ_01_ | 0.53 _PPN_ | | 0.54 _PPN_ | | 0.49 _PPN_ | | 0.61 _PPN_ | | 0.53 _PPN_ | | 0.51 _PPN_ | | 0.55 _PPN_ | | 0.52 _PPN_ | |
| ICC | 0.57 | | 0.51 | | 0.55 | | 0.58 | | 0.57 | | 0.57 | | 0.58 | | 0.57 | |
| N | 40 _PPN_ | | 40 _PPN_ | | 40 _PPN_ | | 40 _PPN_ | | 40 _PPN_ | | 40 _PPN_ | | 40 _PPN_ | | 40 _PPN_ | |
| Observations | 561 | | 561 | | 561 | | 547 | | 561 | | 561 | | 561 | | 561 | |
| Marginal R^2^ / Conditional R^2^ | 0.324 / 0.712 | | 0.397 / 0.704 | | 0.348 / 0.708 | | 0.314 / 0.712 | | 0.324 / 0.713 | | 0.328 / 0.711 | | 0.323 / 0.714 | | 0.326 / 0.712 | |
| AIC | 4987.056 | | 4981.814 | | 4984.801 | | 4868.549 | | 4987.671 | | 4989.665 | | 4990.211 | | 4983.996 | |
| ** p<0.05   ** p<0.01   *** p<0.001* | | | | | | | | | | | | | | | | |

# Table S3

*Model summaries for Hypothesis 1a – CS-*

|  |  | | **USunpleasantness_c** | | **Age_c** | | **Gender** | | **QIDS_c** | | **STAI_c** | | **IUS_c** | | **DTS_c** | |
| --- | --- | --- | --- | --- | --- | --- | --- | --- | --- | --- | --- | --- | --- | --- | --- | --- |
| *Predictors* | *Estimates* | *CI* | *Estimates* | *CI* | *Estimates* | *CI* | *Estimates* | *CI* | *Estimates* | *CI* | *Estimates* | *CI* | *Estimates* | *CI* | *Estimates* | *CI* |
| (Intercept) | 33.20 ^***^ | 25.94 – 40.46 | 33.20 ^***^ | 26.57 – 39.83 | 33.20 ^***^ | 26.32 – 40.08 | 38.29 ^***^ | 28.69 – 47.88 | 33.20 ^***^ | 26.10 – 40.29 | 33.20 ^***^ | 26.00 – 40.39 | 33.20 ^***^ | 25.95 – 40.45 | 33.20 ^***^ | 25.95 – 40.45 |
| Phase1 | 2.58 ^***^ | 1.15 – 4.01 | 2.58 ^***^ | 1.15 – 4.01 | 2.58 ^***^ | 1.15 – 4.01 | 2.48 ^***^ | 1.01 – 3.94 | 2.58 ^***^ | 1.16 – 4.01 | 2.58 ^***^ | 1.16 – 4.01 | 2.58 ^***^ | 1.15 – 4.01 | 2.58 ^***^ | 1.15 – 4.01 |
| Trial c | -3.24 ^***^ | -4.11 – -2.37 | -3.24 ^***^ | -4.11 – -2.37 | -3.24 ^***^ | -4.10 – -2.37 | -3.25 ^***^ | -4.14 – -2.36 | -3.24 ^***^ | -4.11 – -2.37 | -3.24 ^***^ | -4.11 – -2.37 | -3.24 ^***^ | -4.11 – -2.37 | -3.24 ^***^ | -4.11 – -2.37 |
| SHAPS c | -1.77 ^*^ | -3.32 – -0.23 | -1.01 | -2.52 – 0.50 | -2.21 ^**^ | -3.72 – -0.70 | -2.25 ^**^ | -3.95 – -0.55 | -2.22 ^**^ | -3.85 – -0.58 | -2.02 ^*^ | -3.67 – -0.38 | -1.73 ^*^ | -3.33 – -0.13 | -1.82 ^*^ | -3.38 – -0.25 |
| Trial c × SHAPS c | 0.05 | -0.14 – 0.23 | 0.05 | -0.14 – 0.23 | 0.05 | -0.14 – 0.23 | 0.04 | -0.15 – 0.24 | 0.05 | -0.14 – 0.23 | 0.05 | -0.14 – 0.23 | 0.05 | -0.14 – 0.23 | 0.05 | -0.14 – 0.23 |
| covariate |  |  | 0.52 ^**^ | 0.16 – 0.88 | -2.59 ^*^ | -4.78 – -0.41 |  |  | 1.39 | -0.60 – 3.37 | 0.28 | -0.39 – 0.96 | -0.05 | -0.59 – 0.49 | -2.00 | -14.22 – 10.21 |
| covariate1 |  |  |  |  |  |  | -8.11 | -18.13 – 1.91 |  |  |  |  |  |  |  |  |
| **Random Effects** | | | | | | | | | | | | | | | | |
| σ^2^ | 338.50 | | 338.50 | | 338.52 | | 345.34 | | 338.49 | | 338.50 | | 338.50 | | 338.49 | |
| τ_00_ | 524.87 _PPN_ | | 434.72 _PPN_ | | 469.68 _PPN_ | | 507.80 _PPN_ | | 500.92 _PPN_ | | 515.79 _PPN_ | | 524.38 _PPN_ | | 523.73 _PPN_ | |
| τ_11_ | 3.74 _PPN.Trial_c_ | | 3.74 _PPN.Trial_c_ | | 3.73 _PPN.Trial_c_ | | 3.85 _PPN.Trial_c_ | | 3.74 _PPN.Trial_c_ | | 3.74 _PPN.Trial_c_ | | 3.74 _PPN.Trial_c_ | | 3.74 _PPN.Trial_c_ | |
| ρ_01_ | -0.00 _PPN_ | | 0.01 _PPN_ | | -0.16 _PPN_ | | -0.08 _PPN_ | | -0.04 _PPN_ | | 0.00 _PPN_ | | -0.01 _PPN_ | | 0.00 _PPN_ | |
| ICC | 0.62 | | 0.57 | | 0.59 | | 0.60 | | 0.61 | | 0.61 | | 0.62 | | 0.62 | |
| N | 40 _PPN_ | | 40 _PPN_ | | 40 _PPN_ | | 40 _PPN_ | | 40 _PPN_ | | 40 _PPN_ | | 40 _PPN_ | | 40 _PPN_ | |
| Observations | 639 | | 639 | | 639 | | 623 | | 639 | | 639 | | 639 | | 639 | |
| Marginal R^2^ / Conditional R^2^ | 0.130 / 0.666 | | 0.219 / 0.667 | | 0.193 / 0.670 | | 0.156 / 0.666 | | 0.154 / 0.667 | | 0.139 / 0.666 | | 0.130 / 0.666 | | 0.131 / 0.667 | |
| AIC | 5705.132 | | 5701.816 | | 5700.598 | | 5569.783 | | 5703.532 | | 5706.783 | | 5707.836 | | 5701.531 | |
| ** p<0.05   ** p<0.01   *** p<0.001* | | | | | | | | | | | | | | | | |

- - 1. Hypothesis 1b

In the following tables (Table S4-5), each column represents one model with the outcome variable always being the omission SCR. The first column represents the model for testing hypothesis 1b without any covariate being added. The column names on top represents the covariate that was added into the hypothesis testing model, and its effect corresponds to the effect of the predictor ‘covariate’. No anhedonia effect was found either excluding (Table S4) or including (Table S5) SCR non-learners, while the effect of STAI-T remains significant in both cases

# Table S4

*Model summaries for Hypothesis 1b (N = 28, non-learner excluded)*

|  |  | | **USunpleasantness_c** | | **Age_c** | | **Gender** | | **QIDS_c** | | **STAI_c** | | **IUS_c** | | **DTS_c** | |
| --- | --- | --- | --- | --- | --- | --- | --- | --- | --- | --- | --- | --- | --- | --- | --- | --- |
| *Predictors* | *Estimates* | *CI* | *Estimates* | *CI* | *Estimates* | *CI* | *Estimates* | *CI* | *Estimates* | *CI* | *Estimates* | *CI* | *Estimates* | *CI* | *Estimates* | *CI* |
| (Intercept) | -0.23 ^***^ | -0.32 – -0.15 | -0.23 ^***^ | -0.32 – -0.15 | -0.23 ^***^ | -0.32 – -0.15 | -0.20 ^***^ | -0.31 – -0.09 | -0.22 ^***^ | -0.31 – -0.14 | -0.21 ^***^ | -0.29 – -0.13 | -0.22 ^***^ | -0.31 – -0.13 | -0.23 ^***^ | -0.32 – -0.14 |
| CS1 | -0.06 ^**^ | -0.11 – -0.02 | -0.06 ^**^ | -0.11 – -0.02 | -0.06 ^**^ | -0.11 – -0.02 | -0.06 ^**^ | -0.11 – -0.02 | -0.06 ^**^ | -0.11 – -0.02 | -0.06 ^**^ | -0.11 – -0.02 | -0.06 ^**^ | -0.11 – -0.02 | -0.06 ^**^ | -0.11 – -0.02 |
| Trial c | -0.08 ^***^ | -0.10 – -0.06 | -0.08 ^***^ | -0.10 – -0.06 | -0.08 ^***^ | -0.10 – -0.06 | -0.08 ^***^ | -0.10 – -0.06 | -0.08 ^***^ | -0.10 – -0.06 | -0.08 ^***^ | -0.10 – -0.06 | -0.08 ^***^ | -0.10 – -0.06 | -0.08 ^***^ | -0.10 – -0.06 |
| Phase1 | -0.03 | -0.09 – 0.03 | -0.03 | -0.09 – 0.03 | -0.03 | -0.09 – 0.03 | -0.03 | -0.09 – 0.03 | -0.03 | -0.09 – 0.03 | -0.03 | -0.09 – 0.03 | -0.03 | -0.09 – 0.03 | -0.03 | -0.09 – 0.03 |
| SHAPS c | 0.01 | -0.01 – 0.03 | 0.01 | -0.02 – 0.03 | 0.01 | -0.01 – 0.03 | 0.01 | -0.01 – 0.03 | 0.00 | -0.02 – 0.03 | 0.00 | -0.02 – 0.02 | 0.00 | -0.02 – 0.03 | 0.01 | -0.01 – 0.03 |
| CS1 × Trial c | 0.02 ^*^ | 0.00 – 0.04 | 0.02 ^*^ | 0.00 – 0.04 | 0.02 ^*^ | 0.00 – 0.04 | 0.02 ^*^ | 0.00 – 0.04 | 0.02 ^*^ | 0.00 – 0.04 | 0.02 ^*^ | 0.00 – 0.04 | 0.02 ^*^ | 0.00 – 0.04 | 0.02 ^*^ | 0.00 – 0.04 |
| covariate |  |  | -0.00 | -0.01 – 0.00 | -0.00 | -0.03 – 0.02 |  |  | 0.01 | -0.01 – 0.04 | 0.01 ^*^ | 0.00 – 0.02 | 0.00 | -0.00 – 0.01 | -0.03 | -0.19 – 0.14 |
| covariate1 |  |  |  |  |  |  | -0.06 | -0.17 – 0.05 |  |  |  |  |  |  |  |  |
| **Random Effects** | | | | | | | | | | | | | | | | |
| σ^2^ | 0.42 | | 0.42 | | 0.42 | | 0.42 | | 0.42 | | 0.42 | | 0.42 | | 0.42 | |
| τ_00_ | 0.04 _PPN_ | | 0.04 _PPN_ | | 0.04 _PPN_ | | 0.04 _PPN_ | | 0.04 _PPN_ | | 0.03 _PPN_ | | 0.04 _PPN_ | | 0.04 _PPN_ | |
| τ_11_ | 0.02 _PPN.Phase1_ | | 0.02 _PPN.Phase1_ | | 0.02 _PPN.Phase1_ | | 0.02 _PPN.Phase1_ | | 0.02 _PPN.Phase1_ | | 0.01 _PPN.Phase1_ | | 0.02 _PPN.Phase1_ | | 0.02 _PPN.Phase1_ | |
| ρ_01_ | 0.09 _PPN_ | | 0.07 _PPN_ | | 0.08 _PPN_ | | 0.06 _PPN_ | | 0.05 _PPN_ | | 0.04 _PPN_ | | 0.10 _PPN_ | | 0.07 _PPN_ | |
| ICC | 0.11 | | 0.11 | | 0.11 | | 0.11 | | 0.11 | | 0.10 | | 0.11 | | 0.11 | |
| N | 28 _PPN_ | | 40 _PPN_ | | 40 _PPN_ | | 40 _PPN_ | | 40 _PPN_ | | 40 _PPN_ | | 40 _PPN_ | | 40 _PPN_ | |
| Observations | 846 | | 846 | | 846 | | 846 | | 846 | | 846 | | 846 | | 846 | |
| Marginal R^2^ / Conditional R^2^ | 0.075 / 0.181 | | 0.078 / 0.181 | | 0.075 / 0.181 | | 0.078 / 0.180 | | 0.078 / 0.180 | | 0.089 / 0.180 | | 0.077 / 0.181 | | 0.075 / 0.181 | |
| AIC | 1786.600 | | 1797.917 | | 1795.298 | | 1791.523 | | 1794.585 | | 1793.676 | | 1797.469 | | 1791.586 | |
| ** p<0.05   ** p<0.01   *** p<0.001* | | | | | | | | | | | | | | | | |

# Table S5

*Model summaries for Hypothesis 1b (N = 38, the whole sample excluding two SCR non-responders)*

|  |  | | **USunpleasantness_c** | | **Age_c** | | **Gender** | | **QIDS_c** | | **STAI_c** | | **IUS_c** | | **DTS_c** | |
| --- | --- | --- | --- | --- | --- | --- | --- | --- | --- | --- | --- | --- | --- | --- | --- | --- |
| *Predictors* | *Estimates* | *CI* | *Estimates* | *CI* | *Estimates* | *CI* | *Estimates* | *CI* | *Estimates* | *CI* | *Estimates* | *CI* | *Estimates* | *CI* | *Estimates* | *CI* |
| (Intercept) | -0.23 ^***^ | -0.30 – -0.16 | -0.23 ^***^ | -0.30 – -0.17 | -0.23 ^***^ | -0.30 – -0.16 | -0.19 ^***^ | -0.28 – -0.10 | -0.23 ^***^ | -0.30 – -0.16 | -0.23 ^***^ | -0.29 – -0.16 | -0.23 ^***^ | -0.30 – -0.16 | -0.23 ^***^ | -0.30 – -0.16 |
| CS1 | -0.06 ^***^ | -0.10 – -0.03 | -0.06 ^***^ | -0.10 – -0.03 | -0.06 ^***^ | -0.10 – -0.03 | -0.06 ^***^ | -0.10 – -0.03 | -0.06 ^***^ | -0.10 – -0.03 | -0.06 ^***^ | -0.10 – -0.03 | -0.06 ^***^ | -0.10 – -0.03 | -0.06 ^***^ | -0.10 – -0.03 |
| Trial c | -0.07 ^***^ | -0.08 – -0.05 | -0.07 ^***^ | -0.08 – -0.05 | -0.07 ^***^ | -0.08 – -0.05 | -0.07 ^***^ | -0.08 – -0.05 | -0.07 ^***^ | -0.08 – -0.05 | -0.07 ^***^ | -0.08 – -0.05 | -0.07 ^***^ | -0.08 – -0.05 | -0.07 ^***^ | -0.08 – -0.05 |
| Phase1 | -0.03 | -0.09 – 0.03 | -0.03 | -0.09 – 0.03 | -0.03 | -0.09 – 0.03 | -0.03 | -0.08 – 0.03 | -0.03 | -0.09 – 0.03 | -0.03 | -0.09 – 0.03 | -0.03 | -0.09 – 0.03 | -0.03 | -0.09 – 0.03 |
| SHAPS c | 0.01 | -0.01 – 0.02 | 0.00 | -0.01 – 0.02 | 0.01 | -0.01 – 0.02 | 0.01 | -0.01 – 0.02 | 0.01 | -0.01 – 0.02 | 0.00 | -0.01 – 0.02 | 0.00 | -0.01 – 0.02 | 0.01 | -0.01 – 0.02 |
| CS1 × Trial c | 0.03 ^**^ | 0.01 – 0.04 | 0.03 ^**^ | 0.01 – 0.04 | 0.03 ^**^ | 0.01 – 0.04 | 0.03 ^**^ | 0.01 – 0.04 | 0.03 ^**^ | 0.01 – 0.04 | 0.03 ^**^ | 0.01 – 0.04 | 0.03 ^**^ | 0.01 – 0.04 | 0.03 ^**^ | 0.01 – 0.04 |
| covariate |  |  | -0.00 | -0.01 – 0.00 | -0.01 | -0.03 – 0.02 |  |  | 0.01 | -0.01 – 0.03 | 0.01 ^*^ | 0.00 – 0.01 | 0.00 | -0.00 – 0.01 | -0.02 | -0.14 – 0.09 |
| covariate1 |  |  |  |  |  |  | -0.06 | -0.15 – 0.04 |  |  |  |  |  |  |  |  |
| **Random Effects** | | | | | | | | | | | | | | | | |
| σ^2^ | 0.40 | | 0.40 | | 0.40 | | 0.40 | | 0.40 | | 0.40 | | 0.40 | | 0.40 | |
| τ_00_ | 0.03 _PPN_ | | 0.03 _PPN_ | | 0.03 _PPN_ | | 0.03 _PPN_ | | 0.03 _PPN_ | | 0.03 _PPN_ | | 0.03 _PPN_ | | 0.03 _PPN_ | |
| τ_11_ | 0.02 _PPN.Phase1_ | | 0.02 _PPN.Phase1_ | | 0.02 _PPN.Phase1_ | | 0.02 _PPN.Phase1_ | | 0.02 _PPN.Phase1_ | | 0.02 _PPN.Phase1_ | | 0.02 _PPN.Phase1_ | | 0.02 _PPN.Phase1_ | |
| ρ_01_ | 0.16 _PPN_ | | 0.17 _PPN_ | | 0.14 _PPN_ | | 0.12 _PPN_ | | 0.15 _PPN_ | | 0.16 _PPN_ | | 0.15 _PPN_ | | 0.15 _PPN_ | |
| ICC | 0.12 | | 0.12 | | 0.12 | | 0.12 | | 0.12 | | 0.11 | | 0.12 | | 0.12 | |
| N | 38 _PPN_ | | 40 _PPN_ | | 40 _PPN_ | | 40 _PPN_ | | 40 _PPN_ | | 40 _PPN_ | | 40 _PPN_ | | 40 _PPN_ | |
| Observations | 1143 | | 1143 | | 1143 | | 1113 | | 1143 | | 1143 | | 1143 | | 1143 | |
| Marginal R^2^ / Conditional R^2^ | 0.058 / 0.172 | | 0.062 / 0.172 | | 0.058 / 0.172 | | 0.064 / 0.174 | | 0.060 / 0.172 | | 0.067 / 0.172 | | 0.062 / 0.172 | | 0.058 / 0.172 | |
| AIC | 2339.794 | | 2350.896 | | 2348.597 | | 2279.649 | | 2348.603 | | 2347.797 | | 2350.431 | | 2345.468 | |
| ** p<0.05   ** p<0.01   *** p<0.001* | | | | | | | | | | | | | | | | |

## Hypothesis 2

In the following tables (Table S6-8), each column represents one model with the outcome variable always being the avoidance responses. The first column represents the model for testing hypothesis 2 for each CS without any covariate being added. The column names on top represents the covariate that was added into the hypothesis testing model, and its effect corresponds to the effect of the predictor ‘covariate’. Notably, anhedonia effect was only found for the CS+av. In all the other models including covariates, this effect remains significant while none of the other covariate is significant.

# Table S6

*Model summaries for Hypothesis 2 – CS-*

|  |  | | **USunpleasantness_c** | | **Age_c** | | **Gender** | | **QIDS_c** | | **STAI_c** | | **IUS_c** | | **DTS_c** | |
| --- | --- | --- | --- | --- | --- | --- | --- | --- | --- | --- | --- | --- | --- | --- | --- | --- |
| *Predictors* | *Odds Ratios* | *CI* | *Odds Ratios* | *CI* | *Odds Ratios* | *CI* | *Odds Ratios* | *CI* | *Odds Ratios* | *CI* | *Odds Ratios* | *CI* | *Odds Ratios* | *CI* | *Odds Ratios* | *CI* |
| (Intercept) | 0.03 ^***^ | 0.01 – 0.15 | 0.03 ^***^ | 0.01 – 0.15 | 0.03 ^***^ | 0.01 – 0.15 | 0.03 ^**^ | 0.00 – 0.25 | 0.03 ^***^ | 0.01 – 0.15 | 0.03 ^***^ | 0.01 – 0.14 | 0.03 ^***^ | 0.01 – 0.15 | 0.03 ^***^ | 0.01 – 0.15 |
| Trial c | 1.03 | 0.88 – 1.22 | 1.03 | 0.88 – 1.22 | 1.03 | 0.88 – 1.22 | 1.06 | 0.90 – 1.26 | 1.03 | 0.88 – 1.22 | 1.03 | 0.88 – 1.22 | 1.03 | 0.88 – 1.22 | 1.03 | 0.88 – 1.22 |
| SHAPS c | 0.95 | 0.67 – 1.34 | 0.99 | 0.68 – 1.42 | 0.98 | 0.69 – 1.38 | 0.93 | 0.62 – 1.40 | 0.91 | 0.63 – 1.33 | 0.88 | 0.62 – 1.25 | 0.97 | 0.68 – 1.38 | 0.95 | 0.67 – 1.34 |
| Phase1 | 1.04 | 0.71 – 1.52 | 1.04 | 0.71 – 1.52 | 1.04 | 0.71 – 1.52 | 1.00 | 0.68 – 1.47 | 1.04 | 0.71 – 1.52 | 1.04 | 0.71 – 1.52 | 1.04 | 0.71 – 1.52 | 1.04 | 0.71 – 1.52 |
| covariate |  |  | 1.02 | 0.94 – 1.12 | 1.22 | 0.75 – 1.99 |  |  | 1.12 | 0.72 – 1.75 | 1.09 | 0.95 – 1.26 | 0.97 | 0.86 – 1.10 | 0.97 | 0.07 – 14.17 |
| covariate1 |  |  |  |  |  |  | 1.02 | 0.10 – 10.35 |  |  |  |  |  |  |  |  |
| **Random Effects** | | | | | | | | | | | | | | | | |
| σ^2^ | 3.29 | | 3.29 | | 3.29 | | 3.29 | | 3.29 | | 3.29 | | 3.29 | | 3.29 | |
| τ_00_ | 19.73 _PPN_ | | 19.15 _PPN_ | | 18.49 _PPN_ | | 21.97 _PPN_ | | 19.44 _PPN_ | | 17.59 _PPN_ | | 19.56 _PPN_ | | 19.73 _PPN_ | |
| ICC | 0.86 | | 0.85 | | 0.85 | | 0.87 | | 0.86 | | 0.84 | | 0.86 | | 0.86 | |
| N | 40 _PPN_ | | 40 _PPN_ | | 40 _PPN_ | | 40 _PPN_ | | 40 _PPN_ | | 40 _PPN_ | | 40 _PPN_ | | 40 _PPN_ | |
| Observations | 640 | | 640 | | 640 | | 624 | | 640 | | 640 | | 640 | | 640 | |
| Marginal R^2^ / Conditional R^2^ | 0.003 / 0.858 | | 0.011 / 0.855 | | 0.021 / 0.852 | | 0.005 / 0.870 | | 0.010 / 0.857 | | 0.044 / 0.849 | | 0.009 / 0.857 | | 0.003 / 0.857 | |
| AIC | 253.047 | | 254.597 | | 254.095 | | 243.905 | | 254.420 | | 251.896 | | 254.556 | | 255.047 | |
| ** p<0.05   ** p<0.01   *** p<0.001* | | | | | | | | | | | | | | | | |

# Table S7

*Model summaries for Hypothesis 2 – CS+av*

|  |  | | **USunpleasantness_c** | | **Age_c** | | **Gender** | | **QIDS_c** | | **STAI_c** | | **IUS_c** | | **DTS_c** | |
| --- | --- | --- | --- | --- | --- | --- | --- | --- | --- | --- | --- | --- | --- | --- | --- | --- |
| *Predictors* | *Odds Ratios* | *CI* | *Odds Ratios* | *CI* | *Odds Ratios* | *CI* | *Odds Ratios* | *CI* | *Odds Ratios* | *CI* | *Odds Ratios* | *CI* | *Odds Ratios* | *CI* | *Odds Ratios* | *CI* |
| (Intercept) | 25.58 ^***^ | 12.49 – 52.40 | 25.62 ^***^ | 12.52 – 52.43 | 25.90 ^***^ | 12.80 – 52.44 | 24.18 ^***^ | 9.91 – 58.96 | 25.62 ^***^ | 12.50 – 52.52 | 25.59 ^***^ | 12.49 – 52.43 | 25.54 ^***^ | 12.50 – 52.19 | 25.69 ^***^ | 12.54 – 52.62 |
| Trial c | 1.91 ^***^ | 1.58 – 2.31 | 1.92 ^***^ | 1.58 – 2.32 | 1.92 ^***^ | 1.58 – 2.32 | 1.90 ^***^ | 1.57 – 2.31 | 1.91 ^***^ | 1.58 – 2.31 | 1.91 ^***^ | 1.58 – 2.31 | 1.91 ^***^ | 1.58 – 2.31 | 1.91 ^***^ | 1.58 – 2.32 |
| SHAPS c | 1.17 | 1.00 – 1.37 | 1.18 | 1.00 – 1.40 | 1.20 ^*^ | 1.02 – 1.41 | 1.17 | 0.99 – 1.40 | 1.17 | 0.99 – 1.38 | 1.17 | 0.99 – 1.38 | 1.18 ^*^ | 1.00 – 1.38 | 1.17 | 0.99 – 1.37 |
| Phase1 | 1.70 ^***^ | 1.25 – 2.30 | 1.70 ^***^ | 1.25 – 2.30 | 1.70 ^***^ | 1.25 – 2.30 | 1.64 ^**^ | 1.21 – 2.23 | 1.70 ^***^ | 1.25 – 2.30 | 1.70 ^***^ | 1.25 – 2.30 | 1.70 ^***^ | 1.25 – 2.30 | 1.70 ^***^ | 1.25 – 2.30 |
| Trial c × SHAPS c | 1.09 ^***^ | 1.04 – 1.14 | 1.09 ^***^ | 1.04 – 1.14 | 1.09 ^***^ | 1.04 – 1.14 | 1.08 ^***^ | 1.04 – 1.14 | 1.09 ^***^ | 1.04 – 1.14 | 1.09 ^***^ | 1.04 – 1.14 | 1.09 ^***^ | 1.04 – 1.14 | 1.09 ^***^ | 1.04 – 1.14 |
| covariate |  |  | 1.01 | 0.98 – 1.04 | 1.15 | 0.96 – 1.38 |  |  | 1.01 | 0.86 – 1.19 | 1.00 | 0.95 – 1.06 | 0.99 | 0.95 – 1.03 | 0.77 | 0.29 – 2.05 |
| covariate1 |  |  |  |  |  |  | 1.08 | 0.48 – 2.45 |  |  |  |  |  |  |  |  |
| **Random Effects** | | | | | | | | | | | | | | | | |
| σ^2^ | 3.29 | | 3.29 | | 3.29 | | 3.29 | | 3.29 | | 3.29 | | 3.29 | | 3.29 | |
| τ_00_ | 2.43 _PPN_ | | 2.40 _PPN_ | | 2.21 _PPN_ | | 2.52 _PPN_ | | 2.44 _PPN_ | | 2.43 _PPN_ | | 2.39 _PPN_ | | 2.42 _PPN_ | |
| ICC | 0.42 | | 0.42 | | 0.40 | | 0.43 | | 0.43 | | 0.43 | | 0.42 | | 0.42 | |
| N | 40 _PPN_ | | 40 _PPN_ | | 40 _PPN_ | | 40 _PPN_ | | 40 _PPN_ | | 40 _PPN_ | | 40 _PPN_ | | 40 _PPN_ | |
| Observations | 640 | | 640 | | 640 | | 624 | | 640 | | 640 | | 640 | | 640 | |
| Marginal R^2^ / Conditional R^2^ | 0.401 / 0.656 | | 0.405 / 0.656 | | 0.425 / 0.656 | | 0.378 / 0.648 | | 0.401 / 0.656 | | 0.401 / 0.656 | | 0.403 / 0.654 | | 0.404 / 0.657 | |
| AIC | 349.193 | | 351.002 | | 348.301 | | 345.140 | | 351.154 | | 351.160 | | 350.957 | | 350.825 | |
| ** p<0.05   ** p<0.01   *** p<0.001* | | | | | | | | | | | | | | | | |

# Table S8

*Model summaries for Hypothesis 2 – CS+unav*

|  |  | | **USunpleasantness_c** | | **Age_c** | | **Gender** | | **QIDS_c** | | **STAI_c** | | **IUS_c** | | **DTS_c** | |
| --- | --- | --- | --- | --- | --- | --- | --- | --- | --- | --- | --- | --- | --- | --- | --- | --- |
| *Predictors* | *Odds Ratios* | *CI* | *Odds Ratios* | *CI* | *Odds Ratios* | *CI* | *Odds Ratios* | *CI* | *Odds Ratios* | *CI* | *Odds Ratios* | *CI* | *Odds Ratios* | *CI* | *Odds Ratios* | *CI* |
| (Intercept) | 0.87 | 0.46 – 1.67 | 0.87 | 0.46 – 1.67 | 0.87 | 0.44 – 1.72 | 0.59 | 0.28 – 1.23 | 0.87 | 0.45 – 1.69 | 0.87 | 0.47 – 1.64 | 0.87 | 0.45 – 1.67 | 0.87 | 0.46 – 1.67 |
| Trial c | 0.75 ^**^ | 0.61 – 0.91 | 0.75 ^**^ | 0.61 – 0.91 | 0.74 ^**^ | 0.60 – 0.91 | 0.73 ^**^ | 0.59 – 0.91 | 0.75 ^**^ | 0.61 – 0.91 | 0.75 ^**^ | 0.62 – 0.91 | 0.75 ^**^ | 0.61 – 0.91 | 0.75 ^**^ | 0.61 – 0.91 |
| SHAPS c | 0.90 | 0.78 – 1.03 | 0.90 | 0.78 – 1.04 | 0.92 | 0.79 – 1.06 | 0.92 | 0.79 – 1.08 | 0.90 | 0.78 – 1.04 | 0.89 | 0.77 – 1.02 | 0.89 | 0.78 – 1.03 | 0.90 | 0.78 – 1.03 |
| Phase1 | 0.83 | 0.68 – 1.01 | 0.83 | 0.68 – 1.01 | 0.83 | 0.68 – 1.01 | 0.82 | 0.67 – 1.00 | 0.83 | 0.68 – 1.01 | 0.83 | 0.68 – 1.01 | 0.83 | 0.68 – 1.01 | 0.83 | 0.68 – 1.01 |
| Trial c × SHAPS c | 0.96 | 0.92 – 1.00 | 0.96 | 0.92 – 1.00 | 0.96 | 0.92 – 1.00 | 0.96 | 0.91 – 1.00 | 0.96 | 0.92 – 1.00 | 0.96 | 0.92 – 1.00 | 0.96 | 0.92 – 1.00 | 0.96 | 0.92 – 1.00 |
| covariate |  |  | 1.00 | 0.99 – 1.02 | 1.14 ^*^ | 1.03 – 1.27 |  |  | 0.98 | 0.89 – 1.07 | 1.01 | 0.98 – 1.05 | 1.01 | 0.98 – 1.03 | 0.95 | 0.55 – 1.62 |
| covariate1 |  |  |  |  |  |  | 1.80 ^**^ | 1.19 – 2.74 |  |  |  |  |  |  |  |  |
| **Random Effects** | | | | | | | | | | | | | | | | |
| σ^2^ | 3.29 | | 3.29 | | 3.29 | | 3.29 | | 3.29 | | 3.29 | | 3.29 | | 3.29 | |
| τ_00_ | 3.73 _PPN_ | | 3.70 _PPN_ | | 4.06 _PPN_ | | 4.09 _PPN_ | | 3.84 _PPN_ | | 3.44 _PPN_ | | 3.74 _PPN_ | | 3.71 _PPN_ | |
| τ_11_ | 0.29 _PPN.Trial_c_ | | 0.29 _PPN.Trial_c_ | | 0.31 _PPN.Trial_c_ | | 0.32 _PPN.Trial_c_ | | 0.29 _PPN.Trial_c_ | | 0.28 _PPN.Trial_c_ | | 0.29 _PPN.Trial_c_ | | 0.29 _PPN.Trial_c_ | |
| ρ_01_ | 0.98 _PPN_ | | 0.98 _PPN_ | | 1.00 _PPN_ | | 1.00 _PPN_ | | 0.99 _PPN_ | | 0.98 _PPN_ | | 0.98 _PPN_ | | 0.98 _PPN_ | |
| ICC | 0.61 | | 0.61 | |  | |  | | 0.62 | | 0.60 | | 0.61 | | 0.61 | |
| N | 40 _PPN_ | | 40 _PPN_ | | 40 _PPN_ | | 40 _PPN_ | | 40 _PPN_ | | 40 _PPN_ | | 40 _PPN_ | | 40 _PPN_ | |
| Observations | 640 | | 640 | | 640 | | 624 | | 640 | | 640 | | 640 | | 640 | |
| Marginal R^2^ / Conditional R^2^ | 0.096 / 0.651 | | 0.098 / 0.650 | | 0.254 / NA | | 0.266 / NA | | 0.096 / 0.657 | | 0.102 / 0.639 | | 0.097 / 0.652 | | 0.097 / 0.651 | |
| AIC | 690.020 | | 691.798 | | 685.841 | | 661.539 | | 691.742 | | 691.192 | | 691.764 | | 691.969 | |
| ** p<0.05   ** p<0.01   *** p<0.001* | | | | | | | | | | | | | | | | |

## Exploratory analysis 2

# Table S9

*Model summaries for Exploratory analysis 2*

|  | **Averaged avoidance** | |
| --- | --- | --- |
| *Predictors* | *Estimates* | *CI* |
| (Intercept) | 0.51 ^***^ | 0.45 – 0.58 |
| Averaged relief c | 0.00 | -0.00 – 0.00 |
| Phase1 | 0.00 | -0.01 – 0.01 |
| Averaged relief c × Phase1 | -0.00 | -0.00 – 0.00 |
| **Random Effects** | | |
| σ^2^ | 0.00 | |
| τ_00_ _PPN_ | 0.04 | |
| ICC | 0.92 | |
| N _PPN_ | 40 | |
| Observations | 80 | |
| Marginal R^2^ / Conditional R^2^ | 0.024 / 0.924 | |
| AIC | -51.468 | |
| ** p<0.05   ** p<0.01   *** p<0.001* | | |

# Electrical stimulation used in the current study

Table S10

*Electrical stimulation used in the current study*

| **PPN** | **Age** | **Gender** | **Intensity (mA)** | **Rating (0 -10)** |
| --- | --- | --- | --- | --- |
| P01 | 26 | Female | 48 | 7 |
| P02 | 19 | Female | 9 | 8 |
| P03 | 25 | Female | 14 | 8 |
| P04 | 19 | Female | 10 | 9 |
| P05 | 28 | Female | 40 | 7 |
| P06 | 18 | Female | 16 | 8 |
| P07 | 23 | Female | 36 | 8 |
| P08 | 27 | Female | 28 | 8 |
| P09 | 24 | Female | 40 | 7 |
| P10 | 19 | Male | 28 | 7 |
| P11 | 18 | Female | 11 | 9 |
| P12 | 29 | Female | 44 | 9 |
| P14 | 22 | Female | 56 | 10 |
| P15 | 18 | rather not say | 36 | 9 |
| P16 | 26 | Male | 40 | 7 |
| P17 | 19 | Male | 11 | 9 |
| P18 | 20 | Female | 44 | 8 |
| P19 | 22 | Female | 32 | 9 |
| P20 | 20 | Female | 24 | 7 |
| P21 | 23 | Female | 48 | 9.5 |
| P22 | 25 | Female | 22 | 7 |
| P23 | 22 | Male | 36 | 9 |
| P24 | 19 | Female | 32 | 9 |
| P25 | 18 | Female | 15 | 6 |
| P26 | 23 | Female | 52 | 7 |
| P27 | 19 | Male | 56 | 6 |
| P28 | 22 | Female | 40 | 6 |
| P29 | 19 | Female | 28 | 7 |
| P30 | 18 | Female | 32 | 10 |
| P31 | 18 | Male | 24 | 9 |
| P32 | 18 | Male | 36 | 6 |
| P33 | 17 | Female | 36 | 8.5 |
| P34 | 21 | Female | 24 | 9 |
| P35 | 17 | Female | 56 | 8 |
| P36 | 19 | Female | 20 | 8 |
| P37 | 18 | Female | 17 | 8 |
| P38 | 19 | Female | 52 | 9 |
| P39 | 18 | Female | 24 | 7 |
| P40 | 19 | Female | 32 | 10 |
| P41 | 18 | Female | 11 | 8 |


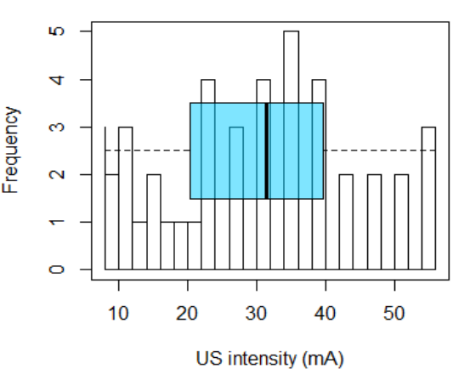

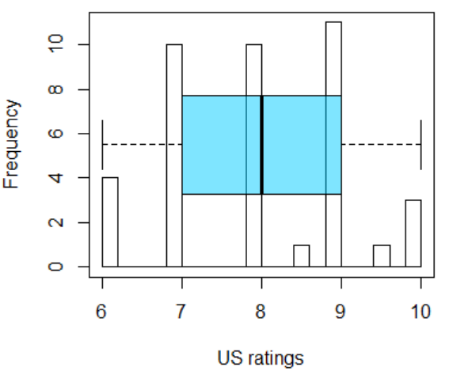


# Trial-by-trial US-expectancy ratings


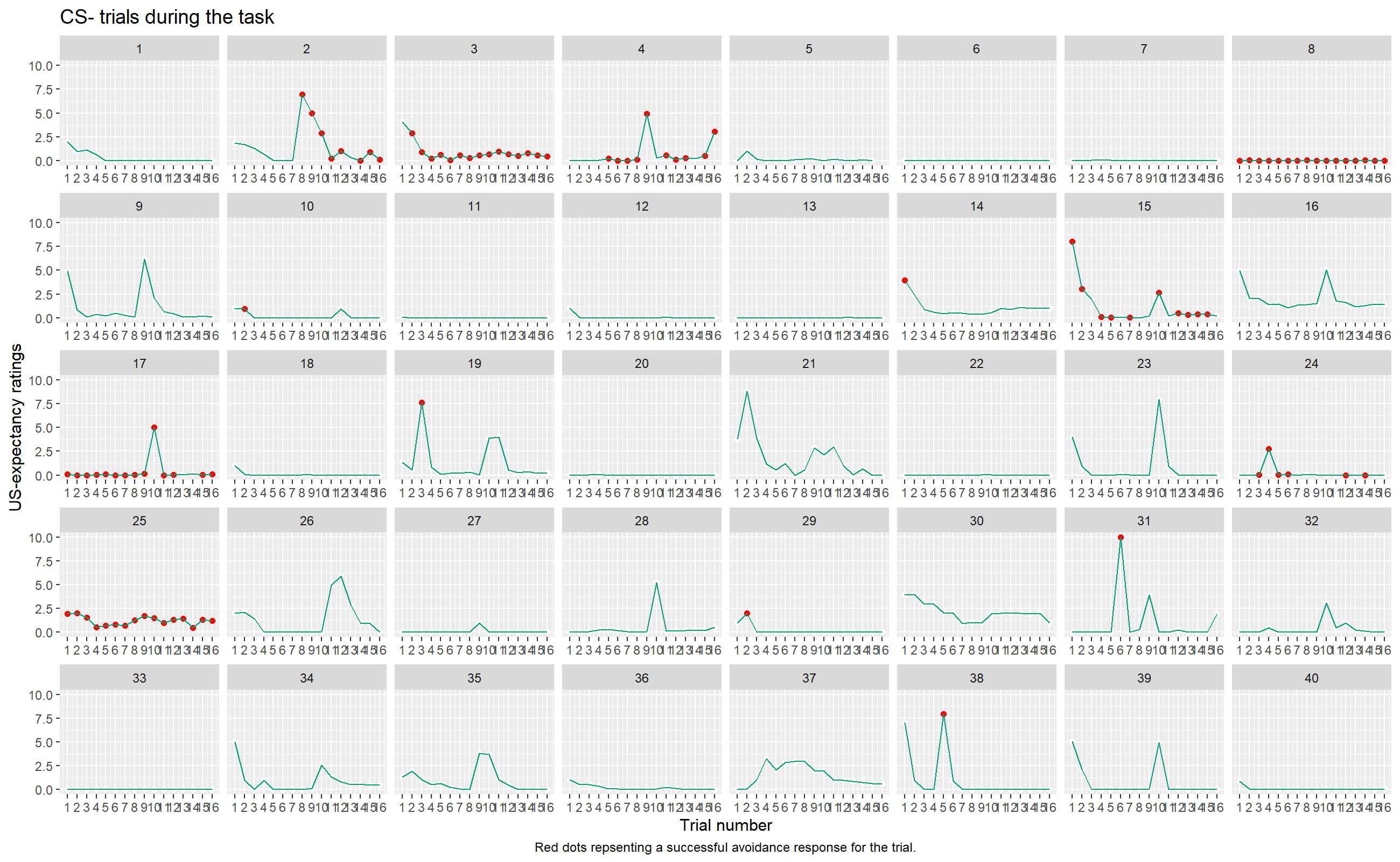


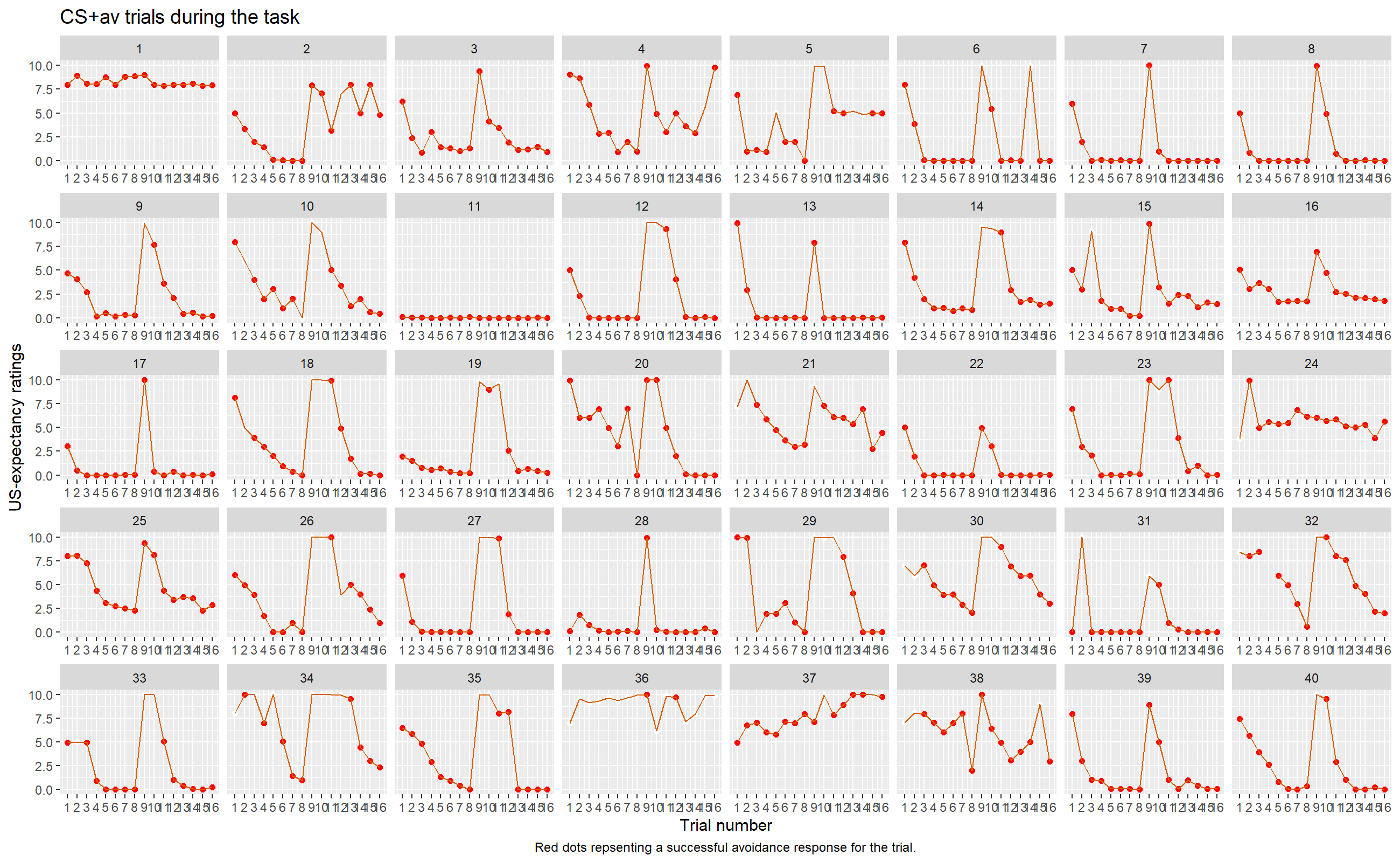


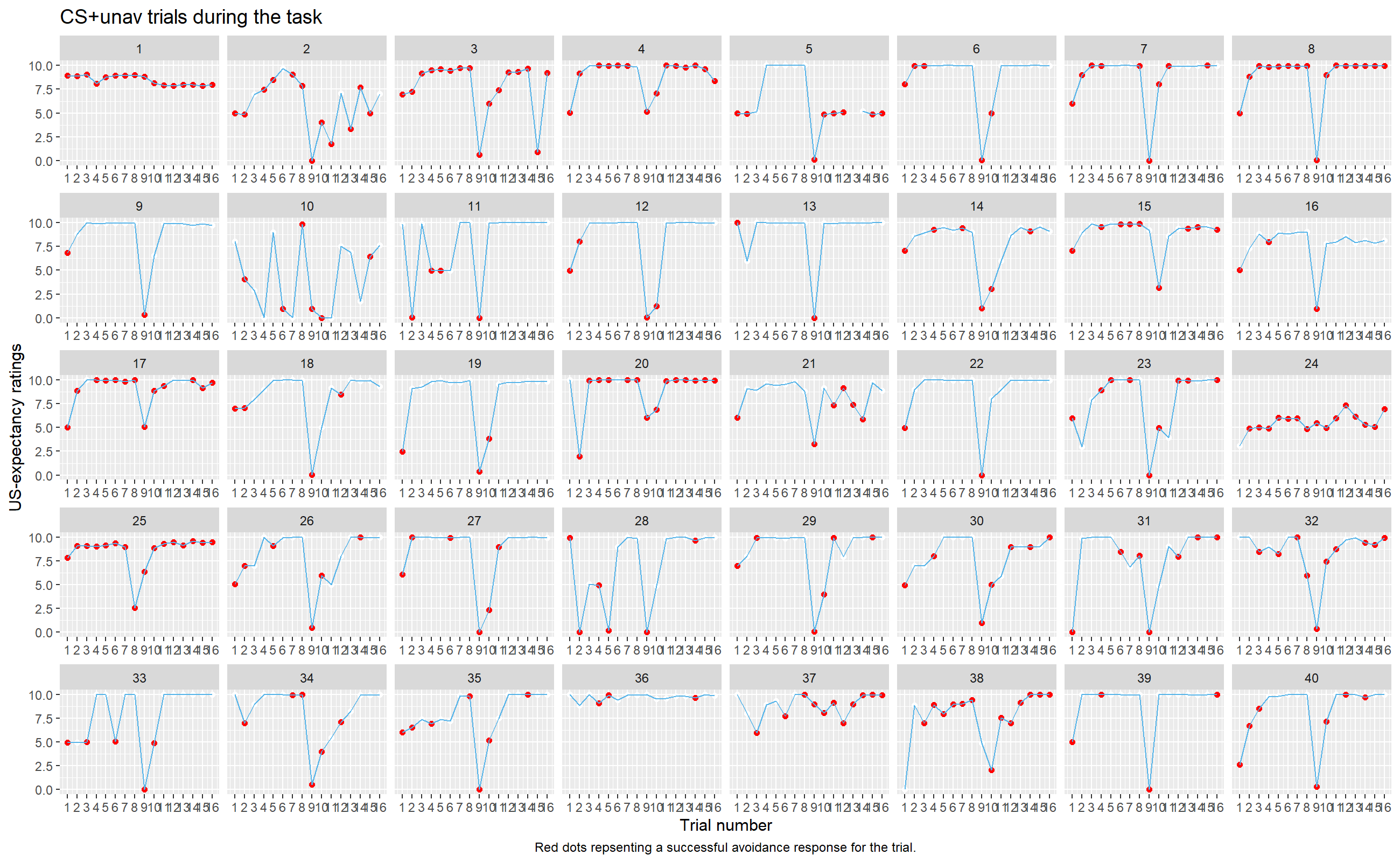


# Trial-by-trial relief-pleasantness ratings


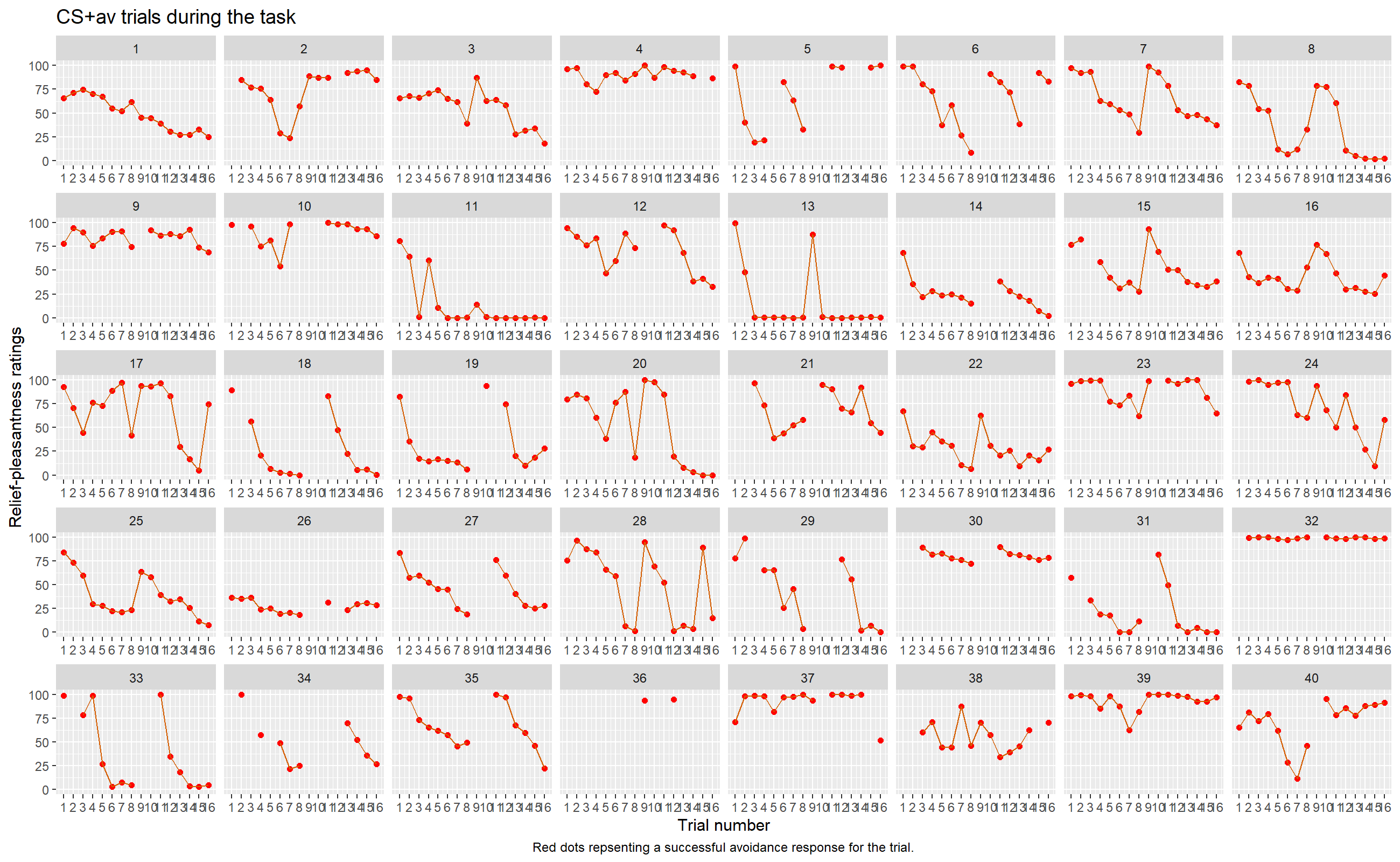


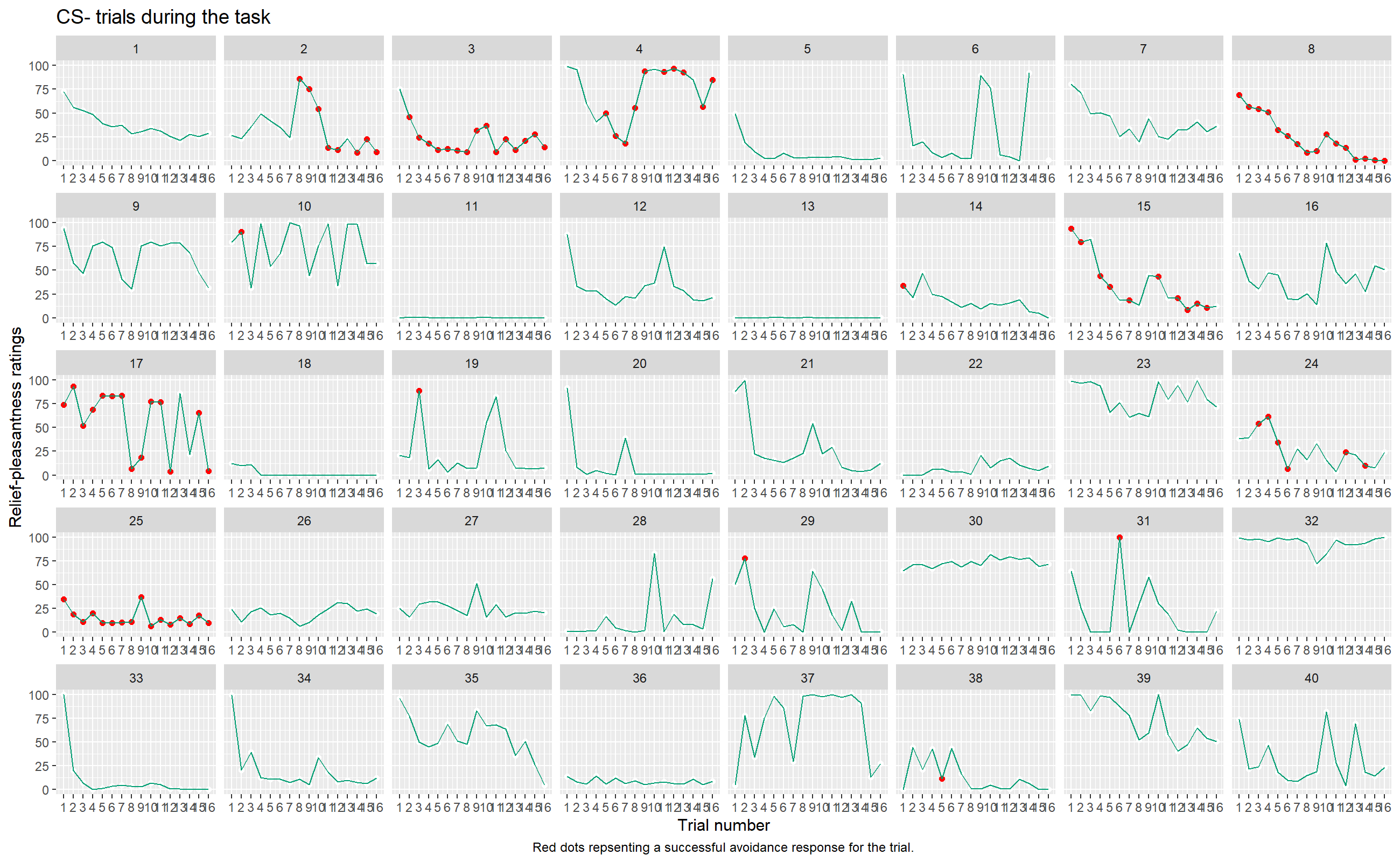


# References

Bates, D., Kliegl, R., Vasishth, S., & Baayen, H. (2018). Parsimonious Mixed Models. *Arxiv:1506.04967[Stat.ME]*. http://arxiv.org/abs/1506.04967

Bates, D., Mächler, M., Bolker, B., & Walker, S. (2014). Fitting Linear Mixed-Effects Models using lme4. *arXiv:1406.5823 [Stat]*. http://arxiv.org/abs/1406.5823

Bickel, R. (2007). *Multilevel Analysis for Applied Research: It’s Just Regression!* Guilford Press.

Brown, V. A. (2021). An Introduction to Linear Mixed-Effects Modeling in R: *Advances in Methods and Practices in Psychological Science*. https://doi.org/10.1177/2515245920960351

Daoud, J. I. (2017). Multicollinearity and Regression Analysis. *Journal of Physics: Conference Series*, *949*, 012009. https://doi.org/10.1088/1742-6596/949/1/012009

Ferron, John M., J. M., Hogarty, K. Y., Dedrick, R. F., Hess, M. R., Niles, J. D., & Kromrey, J. D. (2008). Reporting results from multilevel analyses. In *Multilevel modeling of educational data* (pp. 391–426).

Hartig, F. (2022). *DHARMa: Residual Diagnostics for Hierarchical (Multi-Level / Mixed) Regression Models.* (R package version 0.4.6.) [Computer software]. http://florianhartig.github.io/DHARMa/

Kuznetsova, A., Brockhoff, P. B., & Christensen, R. H. B. (2017). lmerTest Package: Tests in Linear Mixed Effects Models. *Journal of Statistical Software*, *82*(1), Article 1. https://doi.org/10.18637/jss.v082.i13

Luo, W., Li, H., Baek, E., Chen, S., Lam, K. H., & Semma, B. (2021). Reporting Practice in Multilevel Modeling: A Revisit After 10 Years: *Review of Educational Research*. https://doi.org/10.3102/0034654321991229

Russell, V. L. (2021). *Emmeans: Estimated Marginal Means, aka Least-Squares Means. R package version 1.6.1.* [Computer software]. https://CRAN.R-project.org/package=emmeans
